# Rehabilitation and outcomes after complicated vs uncomplicated mild TBI: results from the CENTER-TBI study

**DOI:** 10.1186/s12913-022-08908-0

**Published:** 2022-12-16

**Authors:** Emilie Isager Howe, Marina Zeldovich, Nada Andelic, Nicole von Steinbuechel, Silje C. R. Fure, Ida M. H. Borgen, Marit V. Forslund, Torgeir Hellstrøm, Helene L. Søberg, Unni Sveen, Mari Rasmussen, Ingerid Kleffelgaard, Cathrine Tverdal, Eirik Helseth, Marianne Løvstad, Juan Lu, Juan Carlos Arango-Lasprilla, Olli Tenovuo, Philippe Azouvi, Helen Dawes, Cecilie Roe, CENTER-TBI participants and investigators, Cecilia Åkerlund, Krisztina Amrein, Lasse Andreassen, Audny Anke, Anna Antoni, Gérard Audibert, Maria Luisa Azzolini, Ronald Bartels, Pál Barzó, Romuald Beauvais, Ronny Beer, Bo-Michael Bellander, Antonio Belli, Habib Benali, Maurizio Berardino, Luigi Beretta, Morten Blaabjerg, Peter Bragge, Alexandra Brazinova, Vibeke Brinck, Joanne Brooker, Camilla Brorsson, Andras Buki, Monika Bullinger, Manuel Cabeleira, Alessio Caccioppola, Emiliana Calappi, Maria Rosa Calvi, Peter Cameron, Guillermo Carbayo Lozano, Marco Carbonara, Simona Cavallo, Giorgio Chevallard, Arturo Chieregato, Giuseppe Citerio, Hans Clusmann, Mark Coburn, Jonathan Coles, Jamie D. Cooper, Marta Correia, Amra Čović, Nicola Curry, Endre Czeiter, Marek Czosnyka, Claire Dahyot-Fizelier, Paul Dark, Véronique De Keyser, Vincent Degos, Francesco Della Corte, Hugo den Boogert, Bart Depreitere, Đula Đilvesi, Abhishek Dixit, Emma Donoghue, Jens Dreier, Guy-Loup Dulière, Ari Ercole, Patrick Esser, Erzsébet Ezer, Martin Fabricius, Valery L. Feigin, Kelly Foks, Shirin Frisvold, Alex Furmanov, Pablo Gagliardo, Damien Galanaud, Dashiell Gantner, Guoyi Gao, Pradeep George, Alexandre Ghuysen, Lelde Giga, Ben Glocker, Jagoš Golubovic, Pedro A. Gomez, Johannes Gratz, Benjamin Gravesteijn, Francesca Grossi, Russell L. Gruen, Deepak Gupta, Juanita A. Haagsma, Iain Haitsma, Raimund Helbok, Lindsay Horton, Jilske Huijben, Peter J. Hutchinson, Bram Jacobs, Stefan Jankowski, Mike Jarrett, Ji-yao Jiang, Faye Johnson, Kelly Jones, Mladen Karan, Angelos G. Kolias, Erwin Kompanje, Daniel Kondziella, Evgenios Kornaropoulos, Lars-Owe Koskinen, Noémi Kovács, Ana Kowark, Alfonso Lagares, Linda Lanyon, Steven Laureys, Fiona Lecky, Didier Ledoux, Rolf Lefering, Valerie Legrand, Aurelie Lejeune, Leon Levi, Roger Lightfoot, Hester Lingsma, Andrew I. R. Maas, Ana M. Castaño-León, Marc Maegele, Marek Majdan, Alex Manara, Geoffrey Manley, Costanza Martino, Hugues Maréchal, Julia Mattern, Catherine McMahon, Béla Melegh, David Menon, Tomas Menovsky, Ana Mikolic, Benoit Misset, Visakh Muraleedharan, Lynnette Murray, Ancuta Negru, David Nelson, Virginia Newcombe, Daan Nieboer, József Nyirádi, Otesile Olubukola, Matej Oresic, Fabrizio Ortolano, Aarno Palotie, Paul M. Parizel, Jean-François Payen, Natascha Perera, Vincent Perlbarg, Paolo Persona, Wilco Peul, Anna Piippo-Karjalainen, Matti Pirinen, Dana Pisica, Horia Ples, Suzanne Polinder, Inigo Pomposo, Jussi P. Posti, Louis Puybasset, Andreea Radoi, Arminas Ragauskas, Rahul Raj, Malinka Rambadagalla, Isabel Retel Helmrich, Jonathan Rhodes, Sylvia Richardson, Sophie Richter, Samuli Ripatti, Saulius Rocka, Olav Roise, Jonathan Rosand, Jeffrey V. Rosenfeld, Christina Rosenlund, Guy Rosenthal, Rolf Rossaint, Sandra Rossi, Daniel Rueckert, Martin Rusnák, Juan Sahuquillo, Oliver Sakowitz, Renan Sanchez-Porras, Janos Sandor, Nadine Schäfer, Silke Schmidt, Herbert Schoechl, Guus Schoonman, Rico Frederik Schou, Elisabeth Schwendenwein, Charlie Sewalt, Ranjit D. Singh, Toril Skandsen, Peter Smielewski, Abayomi Sorinola, Emmanuel Stamatakis, Simon Stanworth, Robert Stevens, William Stewart, Ewout W. Steyerberg, Nino Stocchetti, Nina Sundström, Riikka Takala, Viktória Tamás, Tomas Tamosuitis, Mark Steven Taylor, Braden Te Ao, Alice Theadom, Matt Thomas, Dick Tibboel, Marjolein Timmers, Christos Tolias, Tony Trapani, Cristina Maria Tudora, Andreas Unterberg, Peter Vajkoczy, Shirley Vallance, Egils Valeinis, Zoltán Vámos, Mathieu van der Jagt, Gregory Van der Steen, Joukje van der Naalt, Jeroen T. J. M. van Dijck, Inge A. M. van Erp, Thomas A. van Essen, Wim Van Hecke, Caroline van Heugten, Dominique Van Praag, Ernest van Veen, Thijs Vande Vyvere, Roel P. J. van Wijk, Alessia Vargiolu, Emmanuel Vega, Kimberley Velt, Jan Verheyden, Paul M. Vespa, Anne Vik, Rimantas Vilcinis, Victor Volovici, Nicole von Steinbüchel, Daphne Voormolen, Petar Vulekovic, Kevin K. W. Wang, Daniel Whitehouse, Eveline Wiegers, Guy Williams, Lindsay Wilson, Stefan Winzeck, Stefan Wolf, Zhihui Yang, Peter Ylén, Alexander Younsi, Frederick A. Zeiler, Veronika Zelinkova, Agate Ziverte, Tommaso Zoerle

**Affiliations:** 1 Department of Physical Medicine and Rehabilitation, Oslo University Hospital, Oslo, Norway; 2 Institute of Medical Psychology and Medical Sociology, University Medical Center Göttingen, Göttingen, Germany; 3 Faculty of Medicine, Institute of Health and Society, Research Centre for Habilitation and Rehabilitation Models and Services (CHARM), University of Oslo, Oslo, Norway; 4 Department of Psychology, Faculty of Social Sciences, University of Oslo, Oslo, Norway; 5 Faculty of Health Sciences, Oslo Metropolitan University, Oslo, Norway; 6 Department for Occupational Therapy Prosthetics and Orthotics, Faculty of Health Sciences, Oslo Metropolitan University, Oslo, Norway; 7 Department of Neurosurgery, Oslo University Hospital, Oslo, Norway; 8 Faculty of Medicine, Institute of Clinical Medicine, University of Oslo, Oslo, Norway; 9 Research Department, Sunnaas Rehabilitation Hospital, Bjørnemyr, Norway; 10 Department of Family Medicine and Population Health, Division of Epidemiology, Virginia Commonwealth University, Richmond, USA; 11 Department of Psychology, Virginia Commonwealth University, Richmond, USA; 12 Turku Brain Injury Centre, Turku University Hospital, Turku, Finland; 13 Department of Clinical Neurosciences, University of Turku, Turku, Finland; 14 AP-HP, GH Paris-Saclay, Hospital Raymond Poincaré, Garches, France; 15 Université Paris-Saclay, UVSQ, Inserm, CESP, UMR 1018, Team DevPsy, Paris, France; 16 College of Medicine and Health, University of Exeter, Exeter, UK; 17 Oxford Health Biomedical Research Centre, Oxford Health NHS Foundation Trust, Oxford, UK

**Keywords:** Rehabilitation, Mild TBI, PROM

## Abstract

**Background:**

Despite existing guidelines for managing mild traumatic brain injury (mTBI), evidence-based treatments are still scarce and large-scale studies on the provision and impact of specific rehabilitation services are needed. This study aimed to describe the provision of rehabilitation to patients after complicated and uncomplicated mTBI and investigate factors associated with functional outcome, symptom burden, and TBI-specific health-related quality of life (HRQOL) up to six months after injury.

**Methods:**

Patients (*n* = 1379) with mTBI from the Collaborative European NeuroTrauma Effectiveness Research in TBI (CENTER-TBI) study who reported whether they received rehabilitation services during the first six months post-injury and who participated in outcome assessments were included. Functional outcome was measured with the Glasgow Outcome Scale – Extended (GOSE), symptom burden with the Rivermead Post Concussion Symptoms Questionnaire (RPQ), and HRQOL with the Quality of Life after Brain Injury – Overall Scale (QOLIBRI-OS). We examined whether transition of care (TOC) pathways, receiving rehabilitation services, sociodemographic (incl. geographic), premorbid, and injury-related factors were associated with outcomes using regression models. For easy comparison, we estimated ordinal regression models for all outcomes where the scores were classified based on quantiles.

**Results:**

Overall, 43% of patients with complicated and 20% with uncomplicated mTBI reported receiving rehabilitation services, primarily in physical and cognitive domains. Patients with complicated mTBI had lower functional level, higher symptom burden, and lower HRQOL compared to uncomplicated mTBI. Rehabilitation services at three or six months and a higher number of TOC were associated with unfavorable outcomes in all models, in addition to pre-morbid psychiatric problems. Being male and having more than 13 years of education was associated with more favorable outcomes. Sustaining major trauma was associated with unfavorable GOSE outcome, whereas living in Southern and Eastern European regions was associated with lower HRQOL.

**Conclusions:**

Patients with complicated mTBI reported more unfavorable outcomes and received rehabilitation services more frequently. Receiving rehabilitation services and higher number of care transitions were indicators of injury severity and associated with unfavorable outcomes. The findings should be interpreted carefully and validated in future studies as we applied a novel analytic approach.

**Trial registration:**

ClinicalTrials.gov NCT02210221.

**Supplementary Information:**

The online version contains supplementary material available at 10.1186/s12913-022-08908-0.

## Introduction

A high number of patients experience prolonged symptoms, disabilities, and diminished health-related quality of life (HRQOL) after mild traumatic brain injury (mTBI) [1, 2]. In the first six months after injury, patients with complicated mTBI (presence of intracranial injury on computed tomography, CT) report higher symptom burden, poorer functional outcomes and lower HRQOL than those with uncomplicated injury [1, 3–5]. A subgroup of patients with mTBI and extra-cranial injuries also report more symptoms and functional deficits [2]. These patients tend to have longer acute hospital stays and several different care pathways [6] and are often in need of regular follow-up and provision of acute and post-acute rehabilitation services [7]. Substantial geographical variation exists in the organization and provision of acute and post-acute rehabilitation services, which influences outcomes of TBI and challenges comparison of results across studies [8].

Although several guidelines exist on how to manage prolonged symptoms after mTBI, evidence-based treatments are lacking, leading to a risk of inadequate rehabilitation or no rehabilitation at all. In general, there are several challenges in the context of mTBI rehabilitation research, including relatively small sample sizes, and heterogeneity in clinical characteristics, interventions, and outcome measures [9]. A recent systematic review on specialized rehabilitation after mTBI highlighted a need for studies with a larger age range, applying sensitive outcome measures focusing on aspects influenced by mTBI, and assessment at an early stage to identify those with an increased risk of long-term symptoms in need of rehabilitation [10].

This study will use the large patient sample of adults and elderly from the Collaborative European NeuroTrauma Effectiveness Research (CENTER-TBI) study [11] to investigate rehabilitation services provided after mTBI and factors associated with global functioning, symptom burden, and TBI-specific HRQOL six months after injury.

### The specific objectives are to

- Describe transition of care (TOC) pathways and rehabilitation provision to patients after complicated and uncomplicated mTBI in the first six months after the injury.
- Investigate the association between sociodemographic (incl. geographic), premorbid, and injury-related factors, rehabilitation and transitions of care and functional outcome, symptom burden, and TBI-specific HRQOL six months after complicated and uncomplicated mTBI.

Based on the literature, we assume that patients with mTBI, in general, are provided few rehabilitation services. However, we expect that patients with complicated mTBI receive more rehabilitation services than those with uncomplicated mTBI. We also hypothesize that provision of services will be related to the severity of brain injury and overall injury severity, regardless of functional level and symptom burden.

## Methods

### Study design and participants

Participants were recruited between December 19, 2014 and December 17, 2017 within CENTER-TBI, a multicenter, prospective observational longitudinal cohort study conducted in Europe and Israel. The trial was registered at ClinicalTrials.gov on 06.08.2014 (#NCT02210221, https://clinicaltrials.gov/ct2/show/NCT02210221). The core study covered all spectrums of TBI severity (i.e., mild, moderate, and severe) and included *N* = 4509 patients from 65 centers. Included patients had a clinical diagnosis of TBI, an indication for CT scan, and presented to a medical center within 24 h post-injury. Individuals with severe pre-existing neurological disorders were excluded from the core study. First, all patients were evaluated in the emergency room (ER). Then, depending on patients’ needs, three clinical care pathways were differentiated: ER (patients seen in the ER and then discharged), admission to a hospital ward (ADM), or to an intensive care unit (ICU). Further details on the main descriptive findings of CENTER-TBI can be found elsewhere [12].

In the present study, we included *N* = 1379 individuals aged 16 years and above who had sustained a mTBI based on baseline Glasgow Coma Scale (GCS) [13] values (i.e., 13–15). All participants completed the outcome instruments and provided information on receiving rehabilitation services or not within the first six months after injury.

### Ethical approval

The CENTER-TBI study (EC grant 602,150) was conducted in accordance with all relevant laws of the European Union (EU) and all relevant laws of the countries where the recruiting sites were located. Informed consent was obtained from the patients and/or the legal representative/next of kin, according to the local regulations for all participants recruited in the Core Dataset of CENTER-TBI and documented in the electronic case report form (e-CRF). For the full list of sites, ethical committees, and ethical approval details, see the official CENTER-TBI website (https://www.center-tbi.eu/project/ethical-approval).

### Data and instruments

#### Sociodemographic, premorbid, and injury-related data (independent variables)

Sociodemographic and injury-related data were collected at the time of study enrollment. Data comprised the following variables: *sex* (female or male), *age* (continuous and dichotomized at median value), *education* (continuous, i.e., in years, and dichotomized at median value), *living situation* (living alone or not alone), *work participation* (employed, unemployed, and others, i.e., retired, studying, or homemaker).

The *geographical region* was determined based on the country of the participating sites using the EU Vocabularies classification (EuroVoc) [14]. Austria, Belgium, France, Germany, the Netherlands, and the United Kingdom were grouped into *Western European* countries; Italy and Spain into *Southern* region; Denmark, Finland, Latvia, Lithuania, Norway, and Sweden into *Northern* region; Hungary, Romania, and Serbia represented *Eastern Europe*. Southern and Eastern European regions were collapsed into one group due to the small number of participants. Because we were mainly interested in assessing rehabilitation and outcomes across Europe, we limited the analyses to European countries.

The *premorbid somatic health status* (healthy, mild systemic disease, severe systemic disease, and severe systemic disease with constant threat to life) was determined based on the classification of the American Society of Anesthesiologists Physical Status Classification System [15]. Due to a low number of cases, both severe groups were collapsed into one (i.e., severe systematic disease). Premorbid psychological problems were self-reported by the participants (yes/no).

Injury-related data included *injury mechanism* (road traffic accident, fall, and others), *clinical care pathways* (ER, ADM, and ICU), the most frequent *TOC pathways* (ER, ICU, neurological hospital ward [WN], other ward [WO], rehabilitation [RE], home [HO], nursing home [NS]), *number of TOC*, *TBI severity* as measured by the GCS with additional classification based on information on the presence of any intracranial abnormalities on the first CT scan (uncomplicated mTBI: GCS ≥ 13 and no abnormalities on the CT scan; complicated mTBI: GCS ≥ 13 and visible abnormalities on the CT scan), *brain injury severity* assessed by the Abbreviated Injury Scale [16] (AIS; Brain injury AIS, score ≥ 3 considered as severe intracranial injury), and *extracranial injury severity* as measured by the Injury Severity Score (ISS) [17]. The ISS ranges from 0 (no trauma) to 75 (not survivable), and a score > 15 is considered as major overall trauma [18].

#### Rehabilitation

The information on *rehabilitation* was based on the self-reported rehabilitation services received within the first six months (i.e., either at three or six months) after TBI and distinguished between no rehabilitation (0) and any kind of rehabilitation including in- and out-patient services (1). Additionally, we summarized information on TOC reporting all transitions including information on discharge to a rehabilitation unit.

The *number of rehabilitation services provided* was determined by summation of the self-reported information on rehabilitation provision received in various domains (i.e., occupational therapy, physiotherapy, cognitive rehabilitation, speech therapy, and psychological services). Since multiple answers were allowed, we aggregated this information by distinguishing between three groups: no help provided, help provided at least for one domain, and at least for two domains.

The *timing of rehabilitation* was defined as: *early rehabilitation* (within one to three months after injury) and *late rehabilitation* (later than three months after injury).

#### Outcome instruments (dependent variables)

The *functional recovery status* of the participants was assessed using the Glasgow Outcome Scale – Extended (GOSE) [19]. The GOSE is a clinician-reported outcome instrument evaluating functional recovery after TBI on an eight-point scale (1: dead, 2: vegetative state, 3/4: lower/upper severe disability, 5/6: lower/upper moderate disability, 7/8: lower/upper good recovery). Additionally, information can be obtained from the patients or their proxies using the questionnaire version—GOSE-Q [20]. In the present study, missing information on the GOSE was centrally replaced by values substituted from the GOSE-Q or clinical ratings to avoid data loss. Since the GOSE-Q is not able to differentiate between vegetative state and lower severe disability, GOSE levels 2 and 3 were collapsed into one category (2 or 3). The missing values at six-months outcome assessments were imputed using a multi-state model [21].

The *symptom burden* was quantified using the Rivermead Post-Concussion Symptoms Questionnaire (RPQ) [22]. The RPQ is a patient-reported outcome measure (PROM) using a five-point Likert scale (from 0: not experienced at all to 4: a severe problem) to evaluate 16 post-concussion symptoms during the past 24 h compared with the condition before the accident: headache, dizziness, nausea and/or vomiting, noise sensitivity, sleep disturbance, fatigue, irritability, depression, frustration, forgetfulness and poor memory, poor concentration, slow thinking, blurred vision, light sensitivity, double vision, and restlessness. As advised by King et al. [22], all scores of 1 (indicating that the problem was the same as before the injury) were removed. The RPQ total score ranges from 0 to 64.

The *TBI-related HRQOL* was measured by the Quality of Life after Brain Injury—Overall Scale (QOLIBRI-OS) [23]. The QOLIBRI-OS is a short PROM assessing physical condition, cognition, emotions, daily life and autonomy, social relationships, and current and future prospects. The six items are rated on a five-point Likert scale (from 0: not at all to 4: very). The transformed total score ranges from 0 to 100.

### Statistical analyses

Descriptive analyses were carried out to summarize the characteristics of the patients. We report mean, standard deviation, median, and range for continuous data and absolute and relative frequencies for categorical data. Mann–Whitney U tests for continuous data and Chi-square tests for categorical data were applied for comparative analyses.

The number of missing values in independent variables varied from < 1% (*premorbid somatic health status*) to 15% (*education*). Missing data – assumed as missing at random (MAR) – were imputed using multivariate imputation by chained equations (MICE).

Three independent regression models were applied to estimate the associations between sociodemographic (incl. geographic), premorbid, and injury-related factors and respective outcomes (functional recovery status, symptom burden, and TBI-specific HRQOL). We also performed regression analyses in the subsample of the patients with more severe overall trauma (i.e., ISS > 15) to receive a more accurate picture of the influence of the factors on the outcomes in this subsample.

To allow for easy comparison of the results from the regression analyses, we estimated ordinal regression models for all outcomes grouping the scores based on quantiles (i.e., 25%, 50%, and 75%). This classification resulted in three categories for the GOSE since the 75% quantile represented the maximum of the scale: GOSE ≤ 6 (up to moderate recovery), GOSE = 7 (average good recovery), and GOSE 8 = (full recovery). The quantiles for the RPQ resulted in the following four categories: RPQ = 0 (*no symptom burden*), 0 < RPQ ≤ 6 (*low average symptom burden*), 6 < RPQ ≤ 16 (*high average symptom burden*), and RPQ > 16 (*high symptom burden*). The QOLIBRI-OS had the following four groups: QOLIBRI-OS ≤ 58 (*low HRQOL*), 58 < QOLIBRI-OS ≤ 75 (*low average HRQOL*), 75 < QOLIBRI-OS ≤ 83 (*high average HRQOL*), and QOLIBRI-OS > 83 (*high HRQOL*).

Since the main goal was to find associations between the outcomes and the chosen factors, we did not focus on the validation of the regression models and did not report the goodness of model fit.

All analyses were performed with the R version 4.0.2 [24] using the packages table 1 [25] for descriptive analyses and *mice* [26] for the imputation of the missing values. The significance level was set at 5%.

## Results

### Sample characteristics

**Fig. 1:**
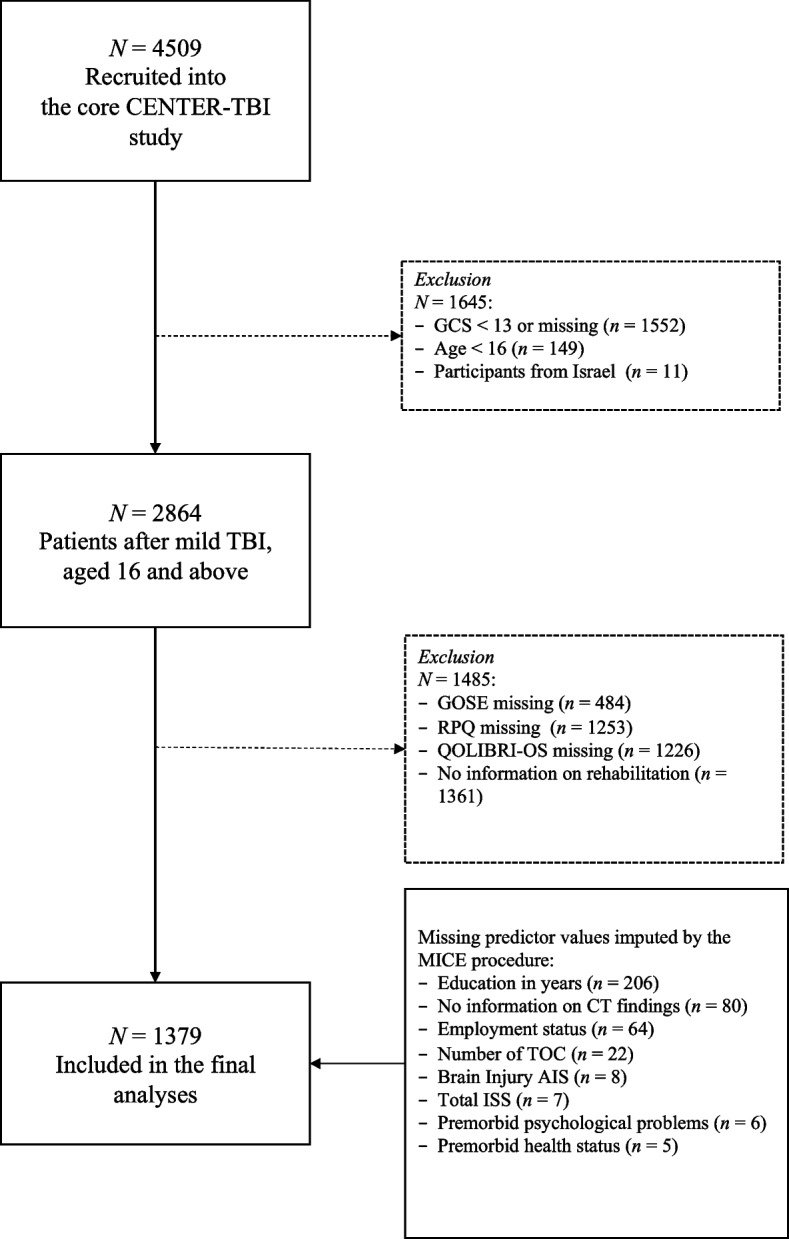
Sample attrition plot

The total sample comprised of 1379 participants (62.9% males, 96% White) with an average age of 52 ± 19 years (*Mdn* = 54, range 16–93). For more details, see the sample attrition plot in Fig. 1.

The distribution of uncomplicated and complicated mTBI was equal (51% and 49%, respectively). Injuries were predominantly caused by falls (47.2%) followed by road traffic accidents (39.3%). At six months after TBI, most individuals (72.9%) showed good recovery (GOSE: 7–8).

The uncomplicated and complicated mTBI group comparisons revealed significant differences regarding age, years of education, employment status, and injury- and rehabilitation-related factors (see Table 1).

**Table 1:** Sociodemographic, premorbid, and injury-related characteristics of the study sample

			mTBI		
		**Total sample**^a^	**Uncomplicated**	**Complicated**	
**Variable**	**Group/value**	**(*N* = 1379)**	**(*N* = 661)**	**(*N* = 638)**	***p*-value**
**Age**	Mean (SD)	52.0 (19.0)	50.0 (18.8)	54.6 (19.1)	** < .001**
	Median [Min, Max]	54.0 [16.0, 93.0]	52.0 [16.0, 89.0]	58.0 [16.0, 93.0]	
**Sex**	Female	512 (37.1%)	262 (39.6%)	219 (34.3%)	0.054
	Male	867 (62.9%)	399 (60.4%)	419 (65.7%)	
**Education years**	< 13	458 (33.2%)	207 (31.3%)	219 (34.3%)	**0.019**
	≥ 13	715 (51.8%)	381 (57.6%)	299 (46.9%)	
	Missing	206 (14.9%)	73 (11.0%)	120 (18.8%)	
**Living situation**	Alone	1103 (80.0%)	529 (80.0%)	506 (79.3%)	0.800
	Not alone	276 (20.0%)	132 (20.0%)	132 (20.7%)	
**Employment status**	Employed	742 (53.8%)	379 (57.3%)	317 (49.7%)	**0.042**
	Unemployed	76 (5.5%)	30 (4.5%)	42 (6.6%)	
	Other	497 (36.0%)	232 (35.1%)	241 (37.8%)	
	Missing	64 (4.6%)	20 (3.0%)	38 (6.0%)	
**Geographical region**	Western Europe	635 (46.0%)	327 (49.5%)	285 (44.7%)	**0.007**
	Northern Europe	386 (28.0%)	184 (27.8%)	159 (24.9%)	
	Southern/Eastern Europe	358 (26.0%)	150 (22.7%)	194 (30.4%)	
**Premorbid physical health status**	Healthy	777 (56.3%)	376 (56.9%)	348 (54.5%)	0.209
	Mild disease	464 (33.6%)	214 (32.4%)	232 (36.4%)	
	Severe disease	133 (9.6%)	70 (10.6%)	55 (8.6%)	
	Missing	5 (0.4%)	1 (0.2%)	3 (0.5%)	
**Premorbid psychological problems**	No	1207 (87.5%)	583 (88.2%)	553 (86.7%)	0.600
	Yes	166 (12.0%)	78 (11.8%)	82 (12.9%)	
	Missing	6 (0.4%)	0 (0%)	3 (0.5%)	
**Injury cause**	Road traffic accident	542 (39.3%)	265 (40.1%)	245 (38.4%)	0.675
	Fall	651 (47.2%)	305 (46.1%)	310 (48.6%)	
	Violent/Other	186 (13.5%)	91 (13.8%)	83 (13.0%)	
**Clinical care pathways**	ER	393 (28.5%)	328 (49.6%)	57 (8.9%)	** < .001**
	ADM	640 (46.4%)	274 (41.5%)	317 (49.7%)	
	ICU	346 (25.1%)	59 (8.9%)	264 (41.4%)	
**Transition of care** **pathways**	ER-HO	370 (26.8%)	320 (48.4%)	42 (6.6%)	n.a
	ER-WN-HO	250 (18.1%)	101 (15.3%)	133 (20.8%)	
	ER-CU-WN-HO	125 (9.1%)	25 (3.8%)	93 (14.6%)	
	ER-WO-HO	116 (8.4%)	75 (11.3%)	37 (5.8%)	
	ER-WARD-HO	77 (5.6%)	44 (6.7%)	28 (4.4%)	
	ER-CU-WO-HO	48 (3.5%)	14 (2.1%)	30 (4.7%)	
	ER-CU-WN-RE	23 (1.7%)	4 (0.6%)	16 (2.5%)	
	ER-CU-CU-WN-HO	18 (1.3%)	4 (0.6%)	13 (2.0%)	
	ER-CU-WN-OT	17 (1.2%)	0 (0%)	15 (2.4%)	
	ER-CU-OT	16 (1.2%)	2 (0.3%)	12 (1.9%)	
	ER-CU-WN-OT-HO	12 (0.9%)	3 (0.5%)	8 (1.3%)	
	ER-WN-OT	11 (0.8%)	1 (0.2%)	9 (1.4%)	
	Other^b^	274 (19.8%)	64 (9.6%)	185 (28.9%)	
	Missing	22 (1.6%)	4 (0.6%)	17 (2.7%)	
**Number of TOC**	Mean (SD)	2.30 (1.28)	1.73 (0.921)	2.84 (1.35)	** < .001**
	Median [Min, Max]	2.00 [1.00, 18.0]	2.00 [1.00, 9.00]	3.00 [1.00, 18.0]	
	Missing	22 (1.6%)	4 (0.6%)	17 (2.7%)	
**Endpoint of TOC**	HO	1190 (86.3%)	630 (95.3%)	500 (78.4%)	n.a
	NH	14 (1.0%)	4 (0.6%)	10 (1.6%)	
	OH	75 (5.4%)	10 (1.5%)	56 (8.8%)	
	RE	75 (5.4%)	12 (1.8%)	53 (8.3%)	
	Other^c^	3 (0.3%)	1 (0.2%)	2 (0.4%)	
	Missing	22 (1.6%)	4 (0.6%)	17 (2.7%)	
**Brain injury AIS**	Mean (SD)	2.54 (1.12)	1.87 (0.904)	3.20 (0.897)	** < .001**
	Median [Min, Max]	3.00 [1.00, 5.00]	2.00 [1.00, 5.00]	3.00 [1.00, 5.00]	
	Missing	8 (0.6%)	1 (0.2%)	6 (0.9%)	
**Total ISS**	Mean (SD)	14.1 (11.5)	9.89 (8.99)	18.3 (12.1)	** < .001**
	Median [Min, Max]	10.0 [1.00, 75.0]	8.00 [1.00, 59.0]	16.0 [1.00, 75.0]	
	Missing	7 (0.5%)	0 (0%)	6 (0.9%)	
**ISS categorized**	No major trauma	863 (62.6%)	531 (80.3%)	288 (45.1%)	** < .001**
	Major trauma	509 (36.9%)	130 (19.7%)	344 (53.9%)	
	Missing	7 (0.5%)	0 (0%)	6 (0.9%)	
**Rehabilitation within six months after TBI**	No rehabilitation	940 (68.2%)	529 (80.0%)	366 (57.4%)	** < .001**
	Rehab. at 3 and/or 6 months	439 (31.8%)	132 (20.0%)	272 (42.6%)	
**GOSE at six months**	Vegetative state/lower severe disability	37 (2.7%)	8 (1.2%)	27 (4.2%)	** < .001**
	Upper severe disability	49 (3.6%)	13 (2.0%)	33 (5.2%)	
	Lower moderate disability	104 (7.5%)	30 (4.5%)	70 (11.0%)	
	Upper moderate disability	184 (13.3%)	60 (9.1%)	111 (17.4%)	
	Lower good recovery	365 (26.5%)	174 (26.3%)	179 (28.1%)	
	Upper good recovery	640 (46.4%)	376 (56.9%)	218 (34.2%)	
**RPQ at six months**	Mean (SD)	10.3 (12.4)	9.05 (12.0)	12.0 (12.8)	** < .001**
	Median [Min, Max]	6.00 [0, 64.0]	4.00 [0, 61.0]	8.00 [0, 64.0]	
**QOLIBRI-OS at six months**	Mean (SD)	69.5 (21.5)	71.1 (21.0)	67.2 (21.7)	** < .001**
	Median [Min, Max]	75.0 [0, 100]	75.0 [0, 100]	71.0 [0, 100]	

Note. *M* Mean, *SD* Standard deviation, *Min* Minimum, *Max* Maximum; n.a.: No comparisons are carried out due to high number of categories and low number of cases; Employment status: employed (full-time employed, part-time employed, on sick leave, special/sheltered employment), unemployed (looking for work, unemployed, unable to work), other (retired, student/school going, homemaker); Geographical regions: Western Europe (Austria, Belgium, France, Germany, the Netherlands, United Kingdom); Northern Europe (Denmark, Finland, Latvia, Lithuania, Norway, Sweden); Southern/Eastern Europe (Italy, Spain, Hungary, Romania, Serbia); *ER* Emergency room, *CU* (high/intensive) Care unit, *WN* Neurological hospital ward, *WO* Other ward, *RE* Rehabilitation, *HO* Home, *NH* Nursing home, *OH* Other hospital, *PSYCH* Psychiatric unit or substance misuse care unit, *AIS* Abbreviated Injury Scale, *ISS* Injury Severity Score; ISS > 15 is considered major trauma; *GOSE* Glasgow Outcome Scale – Extended, *RPQ* Rivermead Post-Concussion Symptoms Questionnaire, *QOLIBRI-OS* Quality of Life After Brain Injury—Overall scale; *p*-values are obtained from the mTBI group comparisons for pairwise complete data (continuous and ordinal variables: Mann–Whitney U-test; categorical variables: chi-square test); significant *p*-values are provided in bold (α = .05)

^a^Due to missing values in the mTBI groups (i.e., missing information on CT findings, *n* = 80, 5.8%), the total sample values (sum and percentage) can exceed the sum between uncomplicated and complicated mTBI frequencies

^b^For transition of care, only pathways containing *n* > 10 are reported, other pathways are collapsed into category “Other”

^c^For the TOC endpoint, only destinations containing *n* > 10 are reported, others are collapsed into category “Other”

### Transition of care (TOC) pathways

**Fig. 2:**
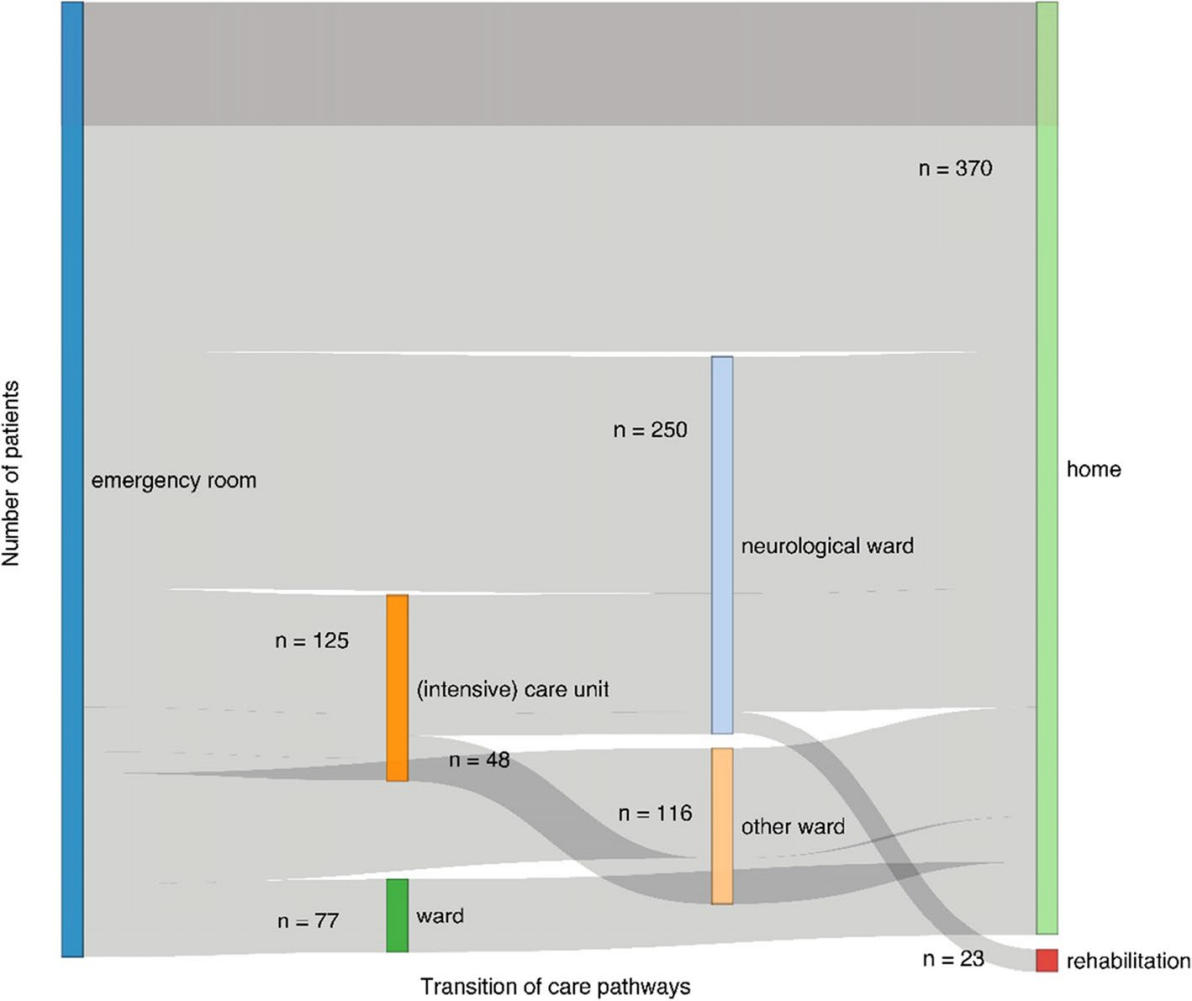
Visualization of the most frequent TOC pathways (*n* > 20). From the starting point (emergency department), patients were either discharged home or transferred to other locations (neurological ward, intensive care unit, or other ward). The end point of the care transition pathways is either discharge to home or admission to a rehabilitation facility. The width of the pathways corresponds to the number of patients in a care transition pathway, with colors representing one pathway

Overall, most of the patients were discharged from the ER back home (26.8%), of whom the majority had sustained an uncomplicated mTBI (85.2%). The average number of TOC differed significantly between the mTBI groups averaging 2 ± 1 in the uncomplicated mTBI group and 3 ± 1 in the complicated mTBI group (W = 89.28, *p* < 0.001). Admission to an inpatient rehabilitation facility at least once during the TOC pathways amounted to 9% of the total sample and 24% for those who reported receiving any kind of rehabilitation during the first half year after TBI. For visualization of the most frequent TOC pathways (*n* > 20), see Fig. 2. Additional visualization of the TOC pathways of the patients with major trauma is provided in Additional file 1 – Figure A1.

### Rehabilitation and provided professional help after mTBI

Rehabilitation services were more frequently provided to patients with complicated mTBI compared to uncomplicated mTBI (42.6% vs. 20.0%). Among those who received any kind of rehabilitation services within six months after mTBI (*n* = 439), 87.9% were treated within the first three months after TBI.

**Fig. 3:**
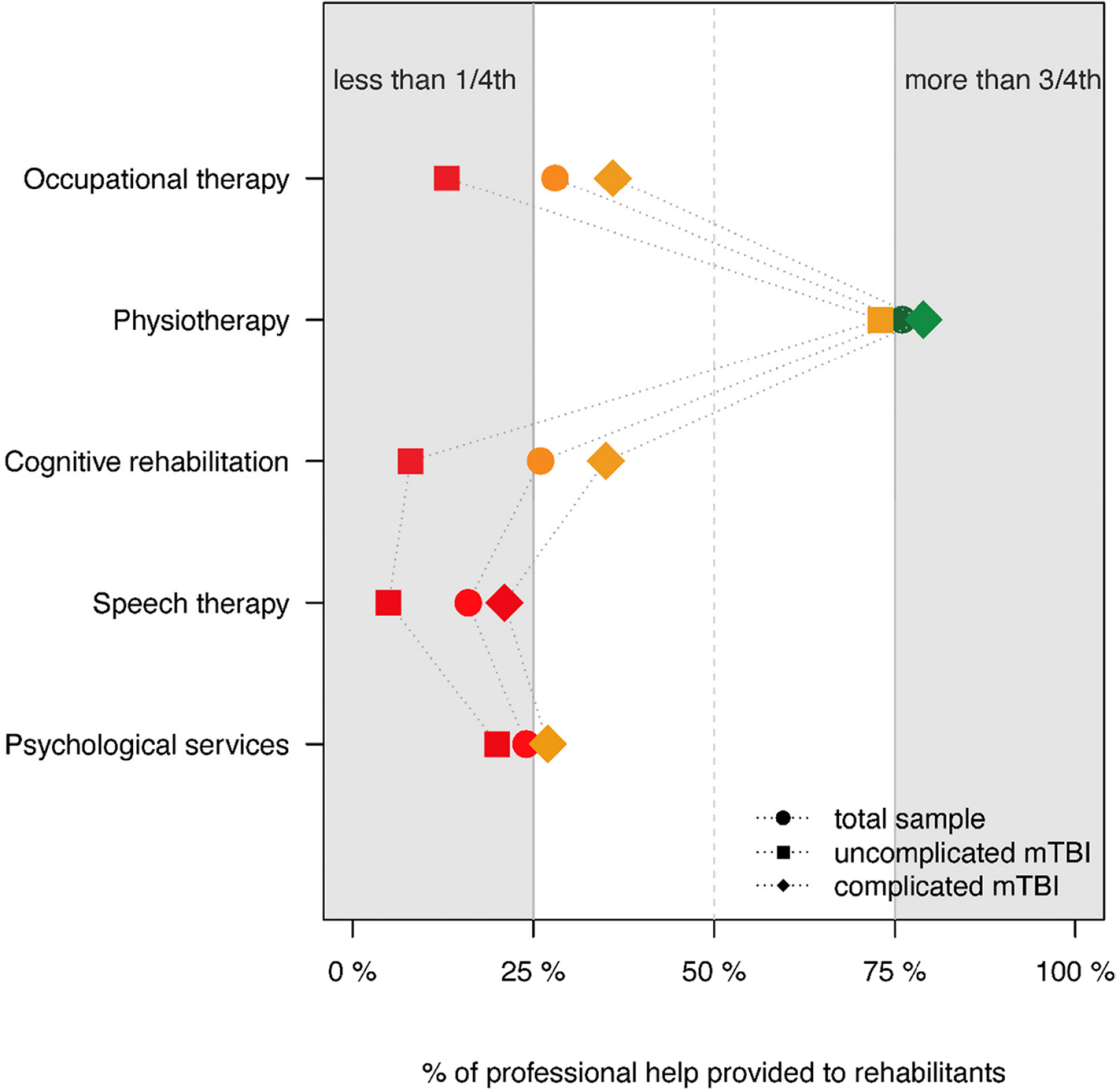
Professional help provided within six months after TBI for rehabilitants. Red symbols indicate professional services provided to less than 25% of patients. Orange symbols indicate that 25% to 75% of rehabilitants received services. Green markers indicate services provided to 75% or more patients

Professional help was provided primarily in physical and cognitive domains. Physical therapy ranked first (76.1%), followed by occupational therapy (27.6%), and cognitive rehabilitation (26.4%). Most of those who received rehabilitation (45.1%) received professional help in more than one domain. The trend was similar between the mTBI groups, with individuals receiving professional help less frequent after uncomplicated mTBI (see Fig. 3). For details on the subsample with major trauma, see Additional file 1 – Figure A2.

### Regression analyses results

#### Functional recovery (GOSE)

**Fig. 4:**
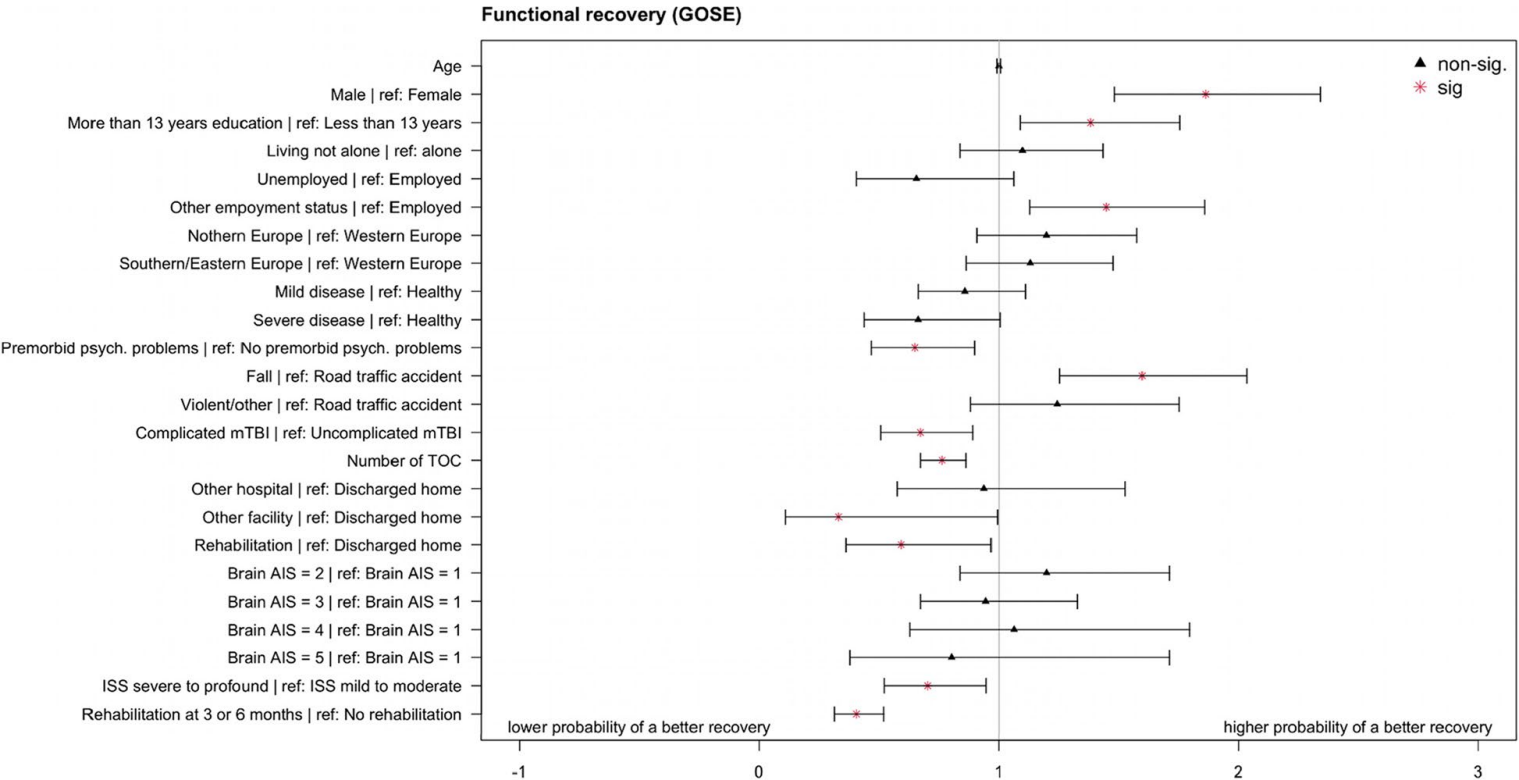
Odds Ratios and 95% CIs for the ordinal logistic regression for the functional recovery status (GOSE)

Higher probability of better functional recovery was associated with the following factors: being male (compared to being female: OR = 1.86, CI_95%_:1.48–2.34), having more than 13 years of education (compared to having less than 13 years of education: OR = 1.38, CI_95%_:1.09–1.76), being retired, student, or homemaker (collapsed into one category called “other” compared to being employed: OR = 1.45, CI_95%_:1.13–1.86), and sustaining a fall (compared to road traffic accident: OR = 1.60, CI_95%_:1.25–2.04). In contrast, the following factors showed lower probability of a better recovery compared to respective reference groups: having premorbid psychological problems (OR = 0.65, CI_95%_:0.47–0.90), sustaining a complicated mTBI (OR = 0.67, CI_95%_:0.51–0.89), having a higher number of TOC (OR = 0.76, CI_95%_:0.67–0.86), being discharged to another facility (OR = 0.33, CI_95%_:0.11–0.99) or being discharged to a rehabilitation facility (OR = 0.59, CI_95%_:0.36–0.97). In addition, sustaining major trauma (OR = 0.70, CI_95%_:0.52–0.95) and receiving rehabilitation (OR = 0.40, CI_95%_:0.31– 0.52) was associated with lower probability of a more favorable recovery. For an overview, see Fig. 4; for more details on regression analyses results, see Additional file 2 – Table A1.

#### Symptom burden (RPQ)

**Fig. 5:**
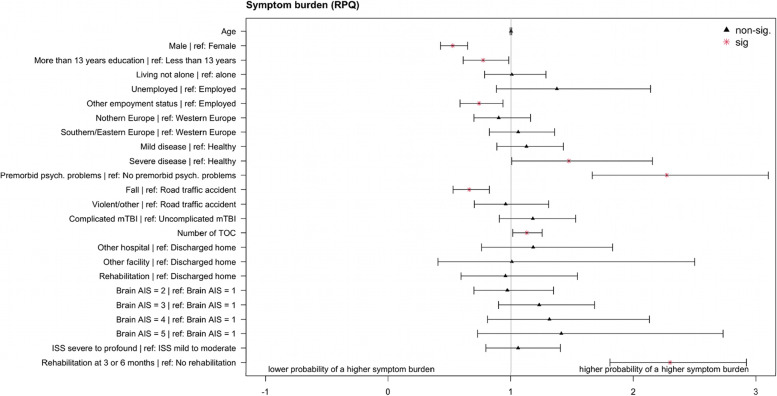
Odds Ratios and 95% CIs for the ordinal logistic regression for the symptom burden groups (RPQ)

The probability of a lower symptom burden was associated with the following factors: being male (OR = 0.52, CI_95%_:0.42–0.65), having more than 13 years of education (OR = 0.77, CI_95%_:0.61–0.98), being retired, student/school going, or homemaker (collapsed into one category called “other”; OR = 0.74, CI_95%_:0.59–0.94), and sustaining a fall (OR = 0.66, CI_95%_:0.53–0.83). The following factors were associated with probability of a higher symptom burden: having a severe premorbid health condition (OR = 1.47, CI_95%_:1.01–2.16), premorbid psychological problems (OR = 2.27, CI_95%_:1.66–3.10), a higher number of TOC (OR = 1.13, CI_95%_:1.01–1.26), and receiving rehabilitation (OR = 2.30, CI_95%_:1.81– 2.92). For an overview, see Fig. 5; for more details on regression analyses results, see Additional file 2 – Table A2.

#### HRQOL (QOLIBRI-OS)

The probability of better HRQOL was associated with the following factors: being male (OR = 1.32, CI_95%_:1.07–1.63) and having more than 13 years of education (OR = 1.36, CI_95%_:1.09–1.70).

**Fig. 6:**
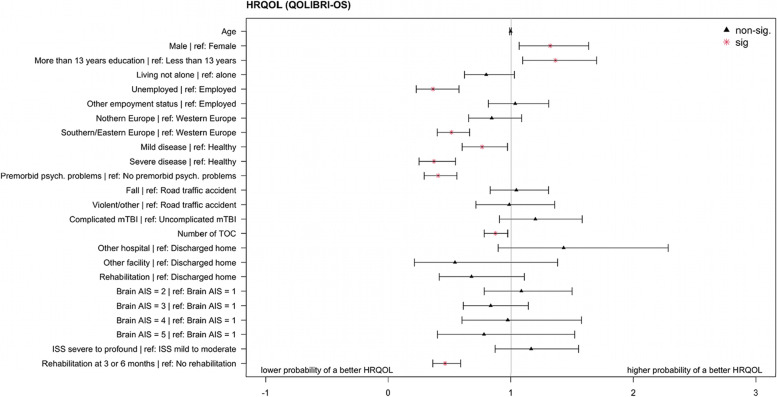
Odds Ratios and 95% CIs for the ordinal logistic regression for the HRQOL groups (QOLIBRI)

In contrast, the following factors showed a lower probability of a higher HRQOL: being unemployed (OR = 0.36, CI_95%_:0.23–0.58), living in Southern or Eastern Europe (OR = 0.51, CI_95%_:0.40–0.66), having a mild (OR = 0.77, CI_95%_:0.60–0.97) or severe health condition prior to TBI (OR = 0.37, CI_95%_:0.25–0.55), suffering from psychological problems before TBI (OR = 0.40, CI_95%_:0.29–0.56), having a higher number of TOC (OR = 0.87, CI_95%_:0.78–0.97), and receiving rehabilitation (OR = 0.46, CI_95%_:0.36– 0.59). For an overview, see Fig. 6; for more details on regression analyses results, see Additional file 2 – Table A3.

Additional analyses in the subsample of the patients with major trauma were carried out for the functional recovery status only as the ISS in the main analyses was associated with lower probability of a better functional outcome. However, the sub-analyses did not find other important associations of interest. For further details, see Additional file 2 – Table A4.

## Discussion

This is the first European study to describe the provision of rehabilitation to patients with complicated and uncomplicated mTBI. Further, to investigate the association between TOC pathways, rehabilitation, sociodemographic (incl. geographic), premorbid, and injury-related factors and functional outcome, symptom burden and TBI-specific HRQL up to 6 months after injury.

Approximately one third of patients with mTBI were discharged from the ER directly to home. The vast majority of these sustained an uncomplicated mTBI. In those admitted to the hospital, the final discharge destination was home as well, and there were few transitions of care to inpatient rehabilitation. Consistent with our first hypothesis, only one fifth of patients with uncomplicated mTBI received rehabilitation services in the first six months after injury. However, for patients with complicated mTBI, the number of care transitions was significantly higher and more complex, showing that 43% received rehabilitation services. The rehabilitation services were primarily provided within the first three months (i.e., acute and sub-acute phase); thus, we could not use this variable in the advanced modeling. The importance of early rehabilitation is, however, assessed in other studies where it has been highlighted that targeted early intervention programs for patients with mTBI might be cost-effective [27].

Physiotherapy was the most frequent service delivered in both patient groups, in line with previous studies [7, 8]. This finding might be related to the fact that physical therapy is the most available service across countries.

The second most frequent services were occupational therapy and cognitive rehabilitation, most frequently delivered to patients with complicated mTBI. In contrast, psychological services were provided to less than one-fourth of the total sample and represented one of the services with the lowest coverage in this study, along with speech therapy. A previous study on all TBI severity levels also reported under-provision of these services [7].

Most of the included cases showed good recovery (GOSE) with low symptom burden (RPQ) and non-impaired HRQOL (QOLIBRI-OS) at 6 months after injury. Nonetheless, we found significantly reduced functional level, higher symptom burden and lower quality of life in complicated mTBI compared to uncomplicated cases. This is in line with previous studies that consider complicated mTBI as a more severe brain injury than uncomplicated mTBI [1, 2, 4]. Indeed, Scandinavian guidelines for initial management of minimal, mild, and moderate TBI [28] recommend that patients with mTBI (GCS 13–15) who have intracranial findings on CT-scans should be managed as patients with moderate TBI. This further highlights the large heterogeneity within the mTBI spectrum.

We found that a higher number of TOC and receiving rehabilitation services was associated with a lower probability of better outcomes. As mentioned previously, higher numbers of TOC and rehabilitation were more frequent in patients with complicated mTBI. Thus, in line with previous studies [6, 29, 30], rehabilitation services provided in this study could be interpreted as an indicator of injury severity.

In addition, sustaining injury due to a fall and more severe injury, as measured by lower GCS score, higher ISS, presence of extracranial injuries and longer acute and rehabilitation hospitalization has previously been found to predict poorer outcomes after TBI [31, 32]. Thus, this finding is unlikely to reflect the effectiveness of rehabilitation services, but rather the nature of the injury. Moreover, poorer outcomes were associated with premorbid somatic and psychological problems. Pre-existing conditions are known to negatively influence outcomes after mTBI [33, 34]. While sustaining a mTBI may exacerbate underlying health conditions, it is also possible that the additive effects of a TBI and existing somatic or psychological problems further complicate the recovery process.

Male gender and higher education was associated with a higher probability of better outcomes, in line with other studies [33–35]. These findings may indicate that individuals at risk of developing incomplete recovery after mTBI, such as females and those with lower education should be offered regular follow-up programs.

Interestingly, in the modeling of HRQOL, we found that residency in Southern/Eastern Europe versus Western Europe was associated with a lower probability of better HRQOL. Whether this finding is due to differences in TBI care across the geographical regions is unclear and the result should be interpreted with caution. Previous reports from this project, however, have highlighted substantial variations in TBI care [36] across Europe that might influence patient outcomes. The results showing a negative association between the Southern/Eastern region and HRQOL are in contrast with a recent health inequalities analysis in Europe that showed the worst health outcomes for participants from the UK (Western Europe) and best outcomes for participants from Italy (Southern Europe) [37]. Analyses based on data obtained from general population samples using the QOLIBRI-OS showed the highest HRQOL reported by Dutch individuals (Western Europe) followed by Italian participants, while respondents from the UK displayed the lowest HRQOL [38].

A previous CENTER-TBI study found that living in Central and Eastern Europe was a significant negative predictor of access to rehabilitation in the first 12 months after TBI [8]. This may reflect the different organization of health care systems in Europe including funding, coverage of rehabilitation services, and referral policies leading to fragmented rehabilitation provision, and serving as important access barriers to rehabilitation after TBI. Future studies are needed to better understand these barriers and their influence on rehabilitation outcomes including HRQOL.

### Strengths and limitations

The present study’s strengths are its large sample size and the high number of participating European countries, which together render a robust overview of mTBI and rehabilitation provision. However, a substantial proportion of trauma centers involved in this study are from urban areas in North-Western Europe. Therefore, the generalizability of findings to other regions is limited.

Further, more than half of patients that received rehabilitation did not provide detailed information on the type of rehabilitation services. In addition, we do not know if rehabilitation services provided in this study were targeted to consequences of TBI or extracranial injuries. Participants were asked to “indicate any help in specific areas that you have been given because of your injury”. Physiotherapy, as the most provided service in this study, is often given due to extracranial injuries.

To avoid loss of statistical power, we imputed missing predictor values using the MICE approach. However, to date, there is no consensus on the goodness-of-fit pooling procedure (e.g., Nagelkerke R^2^) for ordinal logistic regressions with multiply imputed data. Therefore, we relied on the significance of predictors to derive conclusions about the influence of selected factors on outcomes after traumatic brain injury. Our statistical approach allows easy comparisons between differently scaled outcomes, although grouping outcome values by quantiles may result in a loss of information. A methodological extension to assess goodness of fit in ordinal regression models using multiply imputed data would allow a more accurate assessment of model fit.

## Conclusion

Patients with complicated mTBI reported lower functional recovery, higher symptom burden, and lower HRQOL and were more frequently offered rehabilitation services. Received rehabilitation services and higher numbers of care transition pathways as an indicator of injury severity were associated with lower probability of better outcomes in this study. These findings, however, should be interpreted carefully and validated in future studies as we used a novel regression analysis approach for easy comparisons between differently scaled outcome measurements.

## Supplementary Information

**Additional file 1.** Patients with major trauma.

**Additional file 2.** Results of regression analyses.

**Additional file 3.** The CENTER-TBI participants and investigators.

**Additional file 4.** Sites, ethical committees, and ethical approval.

## Data Availability

All relevant data are available upon request from CENTER-TBI, and the authors are not legally allowed to share it publicly. The authors confirm that they received no special access privileges to the data. CENTER-TBI is committed to data sharing and in particular to responsible further use of the data. Hereto, we have a data sharing statement in place: https://www.center-tbi.eu/data/sharing. The CENTER-TBI Management Committee, in collaboration with the General Assembly, established the Data Sharing policy, and Publication and Authorship Guidelines to assure correct and appropriate use of the data as the dataset is hugely complex and requires help of experts from the Data Curation Team or Bio- Statistical Team for correct use. This means that we encourage researchers to contact the CENTER-TBI team for any research plans and the Data Curation Team for any help in appropriate use of the data, including sharing of scripts. Requests for data access can be submitted online: https://www.center-tbi.eu/data. The complete Manual for data access is also available online: https://www.center-tbi.eu/files/SOP-Manual-DAPR-2402020.pdf.

## Abbreviations

ADM: Admission to a hospital ward
AIS: Abbreviated Injury Scale
CENTER-TBI: Collaborative European NeuroTrauma Effectiveness Research in Traumatic Brain Injury
CT: Computed Tomography
e-CFR: Electronic Case Report Form
ER: Emergency Room
GCS: Glasgow Coma Scale
GOSE: Glasgow Outcome Scale – Extended
HO: Home
HRQOL: Health-Related Quality of Life
ICU: Intensive Care Unit
ISS: Injury Severity Score
MAR: Missing At Random
MICE: Multivariate Imputation by Chained Equations
mTBI: Mild Traumatic Brain Injury
NS: Nursing Home
PROM: Patient Reported Outcome Measure
QOLIBRI-OS: Quality of Life After Brain Injury – Overall Scale
RE: Rehabilitation
RPQ: Rivermead Post-Concussion Symptoms Questionnaire
TBI: Traumatic Brain Injury
TOC: Transition of Care
WN: Neurological Hospital Ward
WO: Other Ward
