# Supplementary material for: Rehabilitation and outcomes after complicated vs uncomplicated mild TBI: results from the CENTER-TBI study
Source: BMC Health Serv Res. 2022 Dec 16;22:1536. doi: 10.1186/s12913-022-08908-0 (PMC9758851; doi:10.1186/s12913-022-08908-0)
Supplement: Supplementary file 1 — Additional file 1. Patients with major trauma. [file 12913_2022_8908_MOESM1_ESM.docx]

**Additional file 1 – Patients with major trauma**

**TOC pathways**


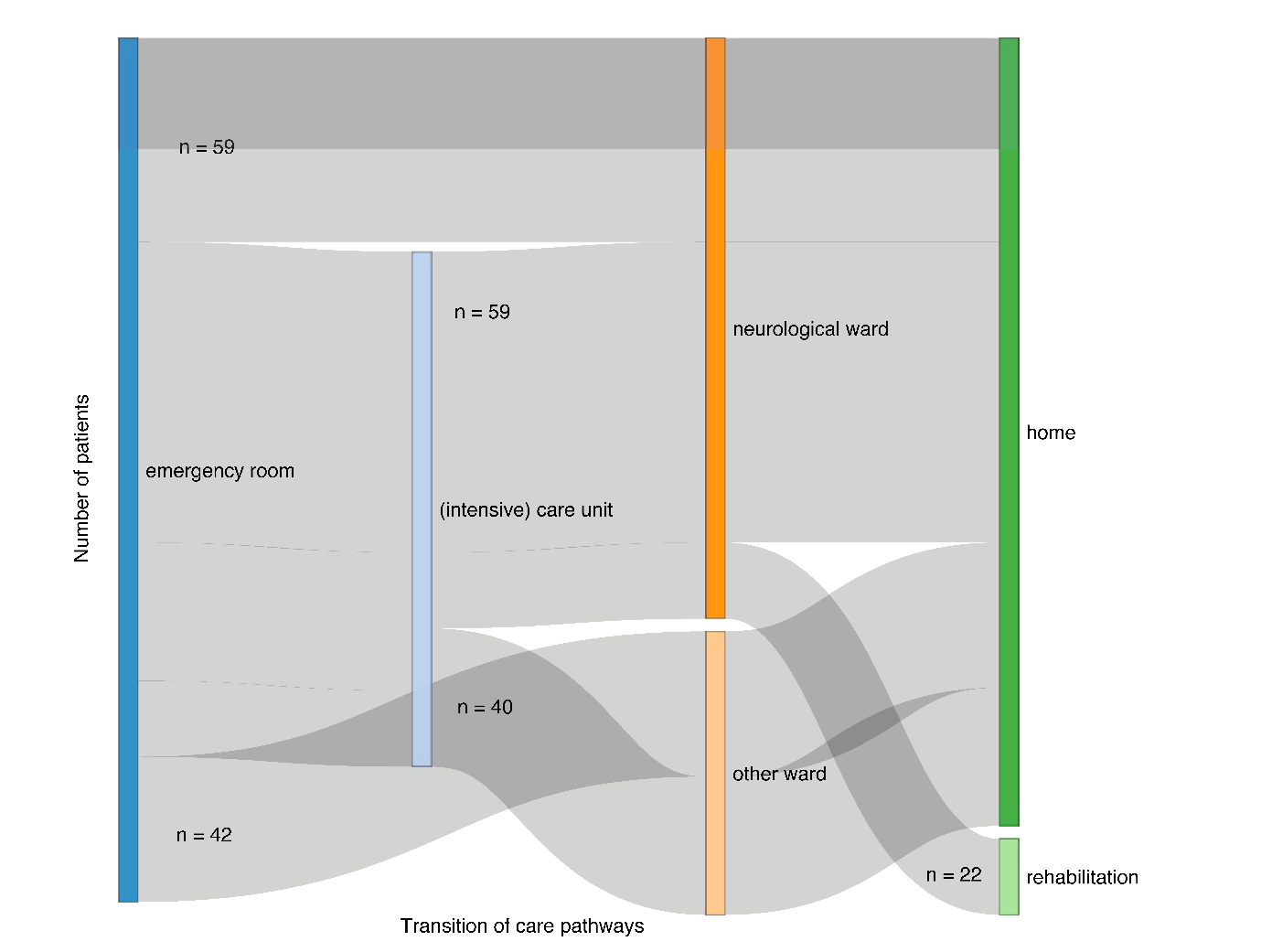


**Figure A1.** Visualization of the most frequent TOC pathways (n > 20) for the patients with major trauma (ISS > 15). The colors indicate one pathway.

**Professional help**

Among those with major trauma (*n* = 509), 50% reported that they received rehabilitation within six months after TBI. Furthermore, 30.3% received professional help in one domain and 31.4% in at least two domains. Professional help was provided primarily in the physical domain (physiotherapy: 52%). All other types of therapy were provided to less than one fourth of the sample. The trend was similar between the mTBI groups, with individuals receiving professional help less frequently after uncomplicated mTBI.


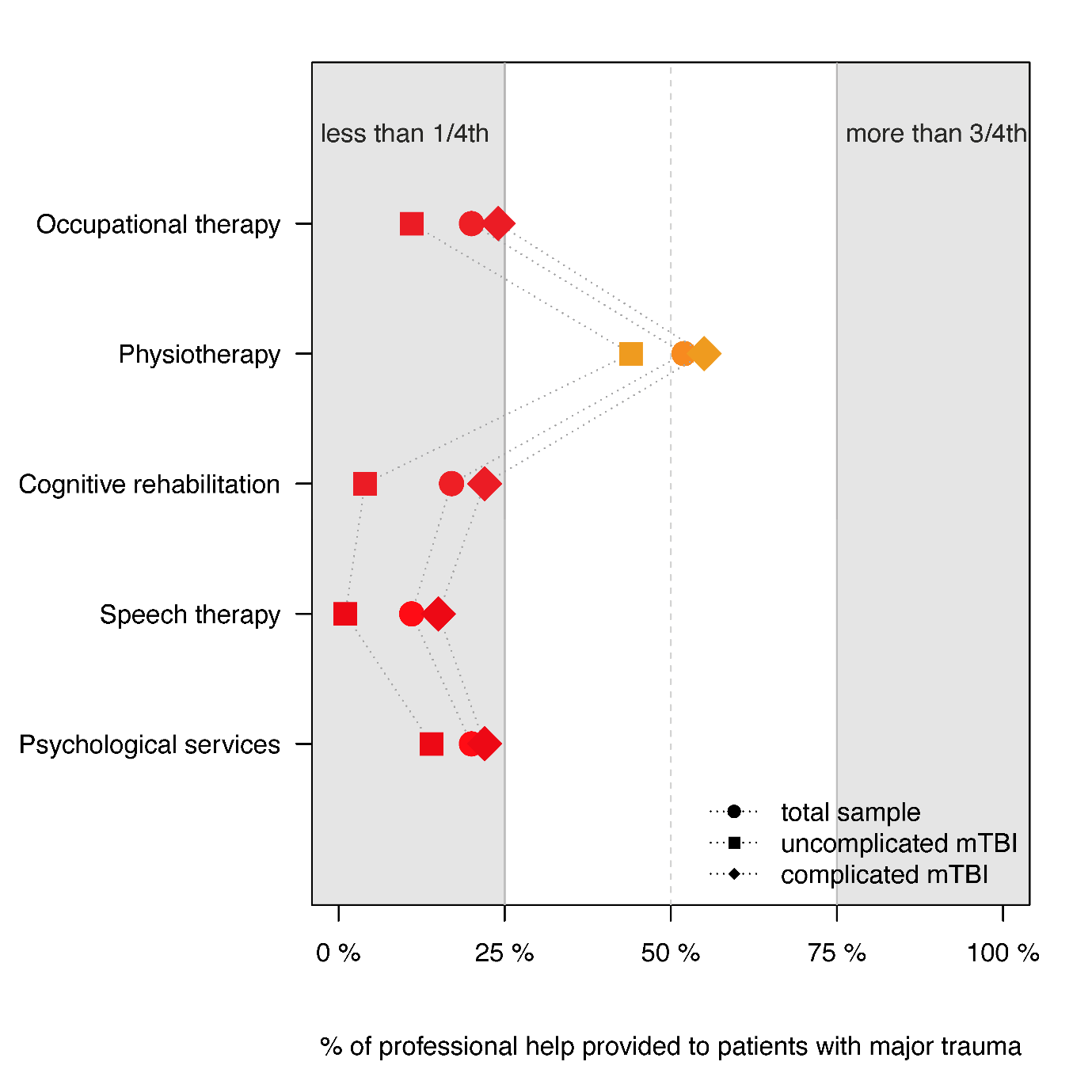


**Figure A2.** Professional help provided within the six months after TBI to all patients with major trauma (ISS > 15), independent of whether they received rehabilitation services. Red symbols indicate professional services provided to less than 25% of patients. Orange symbols indicate that 25% to 75% of rehabilitants received services.
