# Supplementary material for: Rehabilitation and outcomes after complicated vs uncomplicated mild TBI: results from the CENTER-TBI study
Source: BMC Health Serv Res. 2022 Dec 16;22:1536. doi: 10.1186/s12913-022-08908-0 (PMC9758851; doi:10.1186/s12913-022-08908-0)
Supplement: Supplementary file 2 — Additional file 2. Results of regression analyses. [file 12913_2022_8908_MOESM2_ESM.docx]

**Additional file 2 – Results of regression analyses**

**Table A1.** Results of ordinal logistic regression for the functional recovery status (GOSE)

| Factor | Category | Reference | Coefficients | S.E. | *t* | *df* | *p* | OR | CI_2.5%_ | CI_97.5%_ |
| --- | --- | --- | --- | --- | --- | --- | --- | --- | --- | --- |
| Age | Age | - | 0.00 | 0.00 | -0.18 | 1299.47 | 0.858 | 1.00 | 0.99 | 1.01 |
| Sex | Male | Female | 0.62 | 0.12 | 5.35 | 1291.78 | **< 0.001** | 1.86 | 1.48 | 2.34 |
| Education | More than 13 years education | Less than 13 years | 0.32 | 0.12 | 2.68 | 253.39 | **0.008** | 1.38 | 1.09 | 1.76 |
| Living situation | Not alone | Alone | 0.09 | 0.14 | 0.68 | 1344.64 | 0.495 | 1.10 | 0.84 | 1.44 |
| Employment | Unemployed | Employed | -0.42 | 0.25 | -1.72 | 367.39 | 0.086 | 0.66 | 0.41 | 1.06 |
|  | Other | Employed | 0.37 | 0.13 | 2.92 | 1292.36 | **0.004** | 1.45 | 1.13 | 1.86 |
| Geographical region | Northern Europe | Western Europe | 0.18 | 0.14 | 1.29 | 1346.04 | 0.196 | 1.20 | 0.91 | 1.58 |
|  | Southern/Eastern Europe | Western Europe | 0.12 | 0.14 | 0.90 | 1335.20 | 0.369 | 1.13 | 0.86 | 1.48 |
| Premorbid physical health status | Mild disease | Healthy | -0.15 | 0.13 | -1.16 | 1350.07 | 0.247 | 0.86 | 0.66 | 1.11 |
|  | Severe disease | Healthy | -0.41 | 0.21 | -1.94 | 1299.51 | 0.053 | 0.66 | 0.44 | 1.01 |
| Premorbid psychological problems | Yes | No | -0.43 | 0.17 | -2.58 | 1305.85 | **0.010** | *0.65* | 0.47 | 0.90 |
| Injury cause | Fall | Road traffic accident | 0.47 | 0.12 | 3.80 | 1347.36 | **< 0.001** | 1.60 | 1.25 | 2.04 |
|  | Violent/other | Road traffic accident | 0.22 | 0.17 | 1.25 | 1326.00 | 0.212 | 1.24 | 0.88 | 1.75 |
| TBI | Complicated mTBI | Uncomplicated mTBI | -0.40 | 0.14 | -2.75 | 861.87 | **0.006** | *0.67* | 0.51 | 0.89 |
| Number of TOC | Number of TOC | - | -0.27 | 0.06 | -4.25 | 940.28 | **< 0.001** | *0.76* | 0.67 | 0.86 |
| Endpoint of TOC | Other hospital | Discharged home | -0.06 | 0.25 | -0.25 | 981.41 | 0.799 | 0.94 | 0.58 | 1.53 |
|  | Other facility | Discharged home | -1.10 | 0.56 | -1.98 | 209.39 | **0.049** | *0.33* | 0.11 | 0.99 |
|  | Rehabilitation facility | Discharged home | -0.52 | 0.25 | -2.09 | 1300.69 | **0.037** | *0.59* | 0.36 | 0.97 |
| Brain AIS | Brain AIS = 2 | Brain AIS = 1 | 0.18 | 0.18 | 1.00 | 1346.15 | 0.318 | 1.20 | 0.84 | 1.71 |
|  | Brain AIS = 3 | Brain AIS = 1 | -0.06 | 0.17 | -0.32 | 1253.92 | 0.749 | 0.95 | 0.67 | 1.33 |
|  | Brain AIS = 4 | Brain AIS = 1 | 0.06 | 0.27 | 0.23 | 1163.65 | 0.819 | 1.06 | 0.63 | 1.80 |
|  | Brain AIS = 5 | Brain AIS = 1 | -0.22 | 0.39 | -0.57 | 910.32 | 0.571 | 0.80 | 0.38 | 1.71 |
| ISS | Major trauma | No major trauma | -0.35 | 0.15 | -2.31 | 1344.54 | **0.021** | *0.70* | 0.52 | 0.95 |
| Rehabilitation | rehab. at 3 and/or 6 months | No rehabilitation | -0.91 | 0.13 | -7.09 | 1342.81 | **< 0.001** | *0.40* | 0.31 | 0.52 |
| GOSE | Group 1 \| Group 2 | - | -1.58 | 0.26 | -6.11 | 940.02 | **< 0.001** | - | - | - |
|  | Group 2 \| Group 3 | - | -0.18 | 0.25 | -0.70 | 869.63 | **0.485** | - | - | - |
| Note. Estimate = regression coefficient; S.E. = standard error; p = p-value; OR = odds ratio (OR in *italic* represent coefficients showing significantly lower probability of a better recovery, i.e., < 1); CI 2.5% = lower bound of the 95% confidence interval; CI 97.5% = upper bound of the 95% confidence interval; **bold** p-values are significant at 5% α-level. Cut-off values used for the GOSE score: Group 1 (≤ GOSE 6; up to moderate recovery), Group 2 (GOSE = 7; average good recovery), Group 3 (GOSE = 8; upper good recovery). Higher GOSE group indicates better recovery. | | | | | | | | | | |

**Table A2.** Results of ordinal logistic regression for the symptom burden (RPQ)

| Factor | Category | Reference | Coefficients | S.E. | *t* | *df* | *p* | OR | CI_2.5%_ | CI_97.5%_ |
| --- | --- | --- | --- | --- | --- | --- | --- | --- | --- | --- |
| Age | Age | - | 0.00 | 0.00 | 0.04 | 1338.73 | 0.969 | 1.00 | 0.99 | 1.01 |
| Sex | Male | Female | -0.65 | 0.11 | -6.00 | 1344.43 | **< 0.001** | 0.52 | 0.42 | 0.65 |
| Education | More than 13 years education | Less than 13 years | -0.26 | 0.12 | -2.15 | 75.35 | **0.035** | 0.77 | 0.61 | 0.98 |
| Living situation | Not alone | Alone | 0.01 | 0.13 | 0.05 | 1348.35 | 0.960 | 1.01 | 0.79 | 1.29 |
| Employment | Unemployed | Employed | 0.32 | 0.23 | 1.41 | 634.69 | 0.158 | 1.38 | 0.88 | 2.14 |
|  | Other | Employed | -0.30 | 0.12 | -2.51 | 759.32 | **0.012** | 0.74 | 0.59 | 0.94 |
| Geographical region | Northern Europe | Western Europe | -0.10 | 0.13 | -0.80 | 1336.26 | 0.422 | 0.90 | 0.70 | 1.16 |
|  | Southern/Eastern Europe | Western Europe | 0.06 | 0.13 | 0.45 | 1333.57 | 0.650 | 1.06 | 0.83 | 1.36 |
| Premorbid physical health status | Mild disease | Healthy | 0.12 | 0.12 | 0.98 | 1344.63 | 0.327 | 1.13 | 0.89 | 1.43 |
|  | Severe diseased | Healthy | 0.39 | 0.19 | 2.00 | 1345.26 | **0.046** | *1.47* | 1.01 | 2.16 |
| Premorbid psychological problems | Yes | No | 0.82 | 0.16 | 5.16 | 1346.29 | **< 0.001** | *2.27* | 1.66 | 3.10 |
| Injury cause | Fall | Road traffic accident | -0.41 | 0.11 | -3.63 | 1348.21 | **< 0.001** | 0.66 | 0.53 | 0.83 |
|  | Violent/other | Road traffic accident | -0.04 | 0.16 | -0.27 | 1344.74 | 0.791 | 0.96 | 0.70 | 1.31 |
| TBI | Complicated mTBI | Uncomplicated mTBI | 0.16 | 0.13 | 1.24 | 762.39 | 0.216 | 1.18 | 0.91 | 1.53 |
| Number of TOC | Number of TOC | - | 0.12 | 0.05 | 2.23 | 814.51 | **0.026** | *1.13* | 1.01 | 1.26 |
| Endpoint of TOC | Other hospital | Discharged home | 0.17 | 0.22 | 0.75 | 1301.89 | 0.455 | 1.18 | 0.76 | 1.83 |
|  | Other facility | Discharged home | 0.01 | 0.46 | 0.01 | 726.51 | 0.989 | 1.01 | 0.41 | 2.50 |
|  | Rehabilitation facility | Discharged home | -0.04 | 0.24 | -0.18 | 563.12 | 0.858 | 0.96 | 0.59 | 1.54 |
| Brain AIS | Brain AIS = 2 | Brain AIS = 1 | -0.03 | 0.17 | -0.17 | 1341.83 | 0.862 | 0.97 | 0.70 | 1.35 |
|  | Brain AIS = 3 | Brain AIS = 1 | 0.21 | 0.16 | 1.31 | 1341.22 | 0.192 | 1.23 | 0.90 | 1.68 |
|  | Brain AIS = 4 | Brain AIS = 1 | 0.27 | 0.25 | 1.11 | 1060.23 | 0.267 | 1.31 | 0.81 | 2.13 |
|  | Brain AIS = 5 | Brain AIS = 1 | 0.34 | 0.34 | 1.03 | 1297.90 | 0.305 | 1.41 | 0.73 | 2.73 |
| ISS | Major trauma | No major trauma | 0.06 | 0.14 | 0.39 | 1260.84 | 0.695 | 1.06 | 0.80 | 1.40 |
| Rehabilitation | rehab. at 3 and/or 6 months | No rehabilitation | 0.83 | 0.12 | 6.80 | 1320.33 | **< 0.001** | *2.30* | 1.81 | 2.92 |
| RPQ | Group 1 \| Group 2 | - | -0.86 | 0.24 | -3.63 | 1061.39 | **< 0.001** | - | - | - |
|  | Group 2 \| Group 3 | - | 0.29 | 0.24 | 1.25 | 1097.98 | 0.213 | - | - | - |
|  | Group 3 \| Group 4 | - | 1.38 | 0.24 | 5.80 | 1132.17 | **< 0.001** | - | - | - |
| Note. Estimate = regression coefficient; S.E. = standard error; p = p-value; OR = odds ratio (OR in *italic* represent coefficients showing significantly higher probability of symptom burden, i.e., > 1); CI 2.5% = lower bound of the 95% confidence interval; CI 97.5% = upper bound of the 95% confidence interval; **bold** p-values are significant at 5% α-level. Cut-off values used for the RPQ total score: Group 1 (RPQ = 0; no symptom burden), Group 2: (0 < RPQ ≤ 6; low average symptom burden), Group 3 (6 < RPQ ≤ 16; high average symptom burden), Group 4 (RPQ 4 > 16; high symptom burden). Higher group number indicates higher symptom burden. | | | | | | | | | | |

**Table A3.** Results of ordinal logistic regression for the HRQOL (QOLIBRI-OS)

| Factor | Category | Reference | Coefficients | S.E. | *t* | *df* | *p* | OR | CI_2.5%_ | CI_97.5%_ |
| --- | --- | --- | --- | --- | --- | --- | --- | --- | --- | --- |
| Age | Age | - | 0.00 | 0.00 | -1.21 | 1342.07 | 0.226 | 1.00 | 0.99 | 1.00 |
| Sex | Male | Female | 0.28 | 0.11 | 2.56 | 1323.17 | **0.011** | 1.32 | 1.07 | 1.63 |
| Education | More than 13 years education | Less than 13 years | 0.31 | 0.11 | 2.78 | 350.54 | **0.006** | 1.36 | 1.09 | 1.70 |
| Living situation | Not alone | Alone | -0.22 | 0.13 | -1.75 | 1347.59 | 0.080 | 0.80 | 0.62 | 1.03 |
| Employment | Unemployed | Employed | -1.01 | 0.24 | -4.30 | 1233.59 | **< 0.001** | *0.36* | 0.23 | 0.58 |
|  | Other | Employed | 0.03 | 0.12 | 0.28 | 515.73 | 0.783 | 1.03 | 0.82 | 1.31 |
| Geographical region | Northern Europe | Western Europe | -0.17 | 0.13 | -1.31 | 1347.46 | 0.189 | 0.84 | 0.65 | 1.09 |
|  | Southern/Eastern Europe | Western Europe | -0.67 | 0.13 | -5.12 | 1283.65 | **< 0.001** | *0.51* | 0.40 | 0.66 |
| Premorbid physical health status | Mild disease | Healthy | -0.27 | 0.12 | -2.19 | 1341.16 | **0.029** | *0.77* | 0.60 | 0.97 |
|  | Severe diseased | Healthy | -0.99 | 0.20 | -4.99 | 1241.76 | **< 0.001** | *0.37* | 0.25 | 0.55 |
| Premorbid psychological problems | Yes | No | -0.91 | 0.16 | -5.51 | 1301.12 | **< 0.001** | *0.40* | 0.29 | 0.56 |
| Injury cause | Fall | Road traffic accident | 0.04 | 0.12 | 0.36 | 1342.74 | 0.716 | 1.04 | 0.83 | 1.31 |
|  | Violent/other | Road traffic accident | -0.01 | 0.16 | -0.09 | 1339.96 | 0.928 | 0.99 | 0.72 | 1.36 |
| TBI | Complicated mTBI | Uncomplicated mTBI | 0.18 | 0.14 | 1.29 | 199.56 | 0.199 | 1.20 | 0.91 | 1.58 |
| Number of TOC | Number of TOC | - | -0.14 | 0.06 | -2.42 | 1077.82 | **0.016** | *0.87* | 0.78 | 0.97 |
| Endpoint of TOC | Other hospital | Discharged home | 0.36 | 0.24 | 1.50 | 662.65 | 0.133 | 1.43 | 0.90 | 2.28 |
|  | Other facility | Discharged home | -0.61 | 0.48 | -1.28 | 1169.18 | 0.200 | 0.54 | 0.21 | 1.38 |
|  | Rehabilitation facility | Discharged home | -0.39 | 0.25 | -1.56 | 374.18 | 0.121 | 0.68 | 0.42 | 1.11 |
| Brain AIS | Brain AIS = 2 | Brain AIS = 1 | 0.08 | 0.17 | 0.49 | 1343.54 | 0.627 | 1.08 | 0.78 | 1.50 |
|  | Brain AIS = 3 | Brain AIS = 1 | -0.18 | 0.16 | -1.13 | 1227.77 | 0.259 | 0.84 | 0.61 | 1.14 |
|  | Brain AIS = 4 | Brain AIS = 1 | -0.03 | 0.25 | -0.11 | 1196.48 | 0.913 | 0.97 | 0.60 | 1.58 |
|  | Brain AIS = 5 | Brain AIS = 1 | -0.25 | 0.34 | -0.73 | 1261.29 | 0.466 | 0.78 | 0.40 | 1.52 |
| ISS | Major trauma | No major trauma | 0.15 | 0.15 | 1.03 | 1333.47 | 0.301 | 1.16 | 0.87 | 1.55 |
| Rehabilitation | rehab. at 3 and/or 6 months | No rehabilitation | -0.77 | 0.12 | -6.22 | 1341.55 | **< 0.001** | *0.46* | 0.36 | 0.59 |
| QOLIBRI | QOLIBRI 1 \| QOLIBRI 2 | - | -1.97 | 0.24 | -8.14 | 1158.75 | **< 0.001** | - | - | - |
|  | QOLIBRI 2 \| QOLIBRI 3 | - | -0.28 | 0.24 | -1.16 | 1155.29 | **0.245** | - | - | - |
|  | QOLIBRI 3 \| QOLIBRI 4 | - | 0.34 | 0.24 | 1.45 | 1154.75 | **0.148** | - | - | - |
| Note. Estimate = regression coefficient; S.E. = standard error; p = p-value; OR = odds ratio (OR in *italic* represent coefficients showing significantly lower probability of a better HRQOL, i.e., < 1); CI 2.5% = lower bound of the 95% confidence interval; CI 97.5% = upper bound of the 95% confidence interval; **bold** p-values are significant at 5% α-level. Cut-off values used for the QOLIBRI-OS total score: Group 1 (QOLIBRI 1 ≤ 58; low HRQOL), Group 2 (58 < QOLIBRI ≤ 75; low average HRQOL), Group 3 (75 < QOLIBRI ≤ 83; high average HRQOL), Group 4 (QOLIBRI > 83; high HRQOL). Higher group number indicates better HRQOL. | | | | | | | | | | |

**Table A4.** Results of ordinal logistic regression for the functional recovery status (GOSE) for patients with major trauma (ISS > 15)

| Factor | Category | Reference | Coefficients | S.E. | *t* | *df* | *p* | OR | CI_2.5%_ | CI_97.5%_ |
| --- | --- | --- | --- | --- | --- | --- | --- | --- | --- | --- |
| Age | Age | - | 0.01 | 0.01 | 1.14 | 381.31 | 0.257 | 1.01 | 0.99 | 1.02 |
| Sex | Male | Female | 0.68 | 0.20 | 3.37 | 478.20 | **0.001** | 1.97 | 1.33 | 2.92 |
| Education | More than 13 years education | Less than 13 years | 0.26 | 0.20 | 1.32 | 155.77 | 0.187 | 1.30 | 0.88 | 1.92 |
| Living situation | Not alone | Alone | 0.05 | 0.23 | 0.23 | 443.10 | 0.815 | 1.06 | 0.67 | 1.66 |
| Employment | Unemployed | Employed | -0.85 | 0.45 | -1.89 | 84.96 | 0.063 | *0.43* | 0.18 | 1.05 |
|  | Other | Employed | 0.54 | 0.22 | 2.51 | 293.30 | **0.013** | 1.72 | 1.12 | 2.63 |
| Geographical region | Northern Europe | Western Europe | 0.19 | 0.23 | 0.83 | 475.35 | 0.409 | 1.21 | 0.77 | 1.89 |
|  | Southern/Eastern Europe | Western Europe | 0.17 | 0.22 | 0.79 | 469.59 | 0.429 | 1.19 | 0.77 | 1.84 |
| Premorbid physical health status | Mild disease | Healthy | -0.27 | 0.21 | -1.28 | 464.22 | 0.202 | 0.77 | 0.51 | 1.15 |
|  | Severe disease | Healthy | -0.34 | 0.34 | -1.01 | 464.31 | 0.312 | 0.71 | 0.36 | 1.38 |
| Premorbid psychological problems | Yes | No | -0.84 | 0.31 | -2.70 | 369.63 | **0.007** | *0.43* | 0.23 | 0.80 |
| Injury cause | Fall | Road traffic accident | 0.00 | 0.20 | 0.02 | 474.79 | 0.982 | 1.00 | 0.68 | 1.49 |
|  | Violent/other | Road traffic accident | -0.06 | 0.29 | -0.21 | 456.93 | 0.830 | 0.94 | 0.53 | 1.67 |
| TBI | Complicated mTBI | Uncomplicated mTBI | -0.16 | 0.25 | -0.62 | 165.90 | 0.538 | 0.86 | 0.52 | 1.41 |
| Number of TOC | Number of TOC | - | -0.10 | 0.08 | -1.29 | 404.96 | 0.198 | *0.90* | 0.77 | 1.06 |
| Endpoint of TOC | Other hospital | Discharged home | -0.01 | 0.32 | -0.03 | 357.73 | 0.973 | 0.99 | 0.53 | 1.84 |
|  | Other facility | Discharged home | -0.76 | 0.68 | -1.11 | 62.06 | 0.269 | 0.47 | 0.12 | 1.83 |
|  | Rehabilitation facility | Discharged home | -0.54 | 0.30 | -1.78 | 470.60 | 0.076 | 0.58 | 0.32 | 1.06 |
| Brain AIS | Brain AIS = 2 | Brain AIS = 1 | 0.56 | 0.49 | 1.15 | 476.38 | 0.251 | 1.75 | 0.67 | 4.58 |
|  | Brain AIS = 3 | Brain AIS = 1 | 0.12 | 0.43 | 0.27 | 421.84 | 0.784 | 1.12 | 0.49 | 2.60 |
|  | Brain AIS = 4 | Brain AIS = 1 | 0.04 | 0.45 | 0.08 | 384.45 | 0.936 | 1.04 | 0.42 | 2.54 |
|  | Brain AIS = 5 | Brain AIS = 1 | -0.38 | 0.54 | -0.70 | 422.62 | 0.483 | 0.69 | 0.24 | 1.97 |
| Rehabilitation | rehab. at 3 and/or 6 months | No rehabilitation | -0.65 | 0.19 | -3.32 | 470.36 | **0.001** | *0.52* | 0.36 | 0.77 |
| GOSE | GOSE 1 \| GOSE 2 | - | -0.15 | 0.57 | -0.26 | 436.60 | 0.791 | - | - | - |
|  | GOSE 2 \| GOSE 3 | - | 1.22 | 0.57 | 2.14 | 439.89 | **0.033** | - | - | - |
| Note. Estimate = regression coefficient; S.E. = standard error; p = p-value; OR = odds ratio (OR in *italic* represent coefficients showing significantly lower probability of a better recovery, i.e., < 1); CI 2.5% = lower bound of the 95% confidence interval; CI 97.5% = upper bound of the 95% confidence interval; **bold** p-values are significant at 5% α-level. Cut-off values used for the GOSE score: Group 1 (≤ GOSE 6; up to moderate recovery), Group 2 (GOSE = 7; average good recovery), Group 3 (GOSE = 8; upper good recovery). Higher GOSE group indicates better recovery. | | | | | | | | | | |
