# Supplementary material for: Rehabilitation and outcomes after complicated vs uncomplicated mild TBI: results from the CENTER-TBI study
Source: BMC Health Serv Res. 2022 Dec 16;22:1536. doi: 10.1186/s12913-022-08908-0 (PMC9758851; doi:10.1186/s12913-022-08908-0)
Supplement: Supplementary file 3 — Additional file 3. The CENTER-TBI participants and investigators. [file 12913_2022_8908_MOESM3_ESM.docx]

**Additional file 3 - The CENTER-TBI participants and investigators**:

Cecilia Åkerlund, Karolinska Institutet, Stockholm, Swolvaseden; Krisztina Amrein, University of Pécs, Pécs, Hungary; Nada Andelic, Oslo University Hospital and University of Oslo, Oslo, Norway; Lasse Andreassen, University Hospital Northern Norway, Tromso, Norway; Audny Anke, University Hospital Northern Norway, Tromso, Norway; Anna Antoni, Medical University Vienna, Vienna, Austria; Gérard Audibert, University Hospital Nancy, Nancy, France; Philippe Azouvi; Raymond Poincare Hospital, Assistance Publique – Hopitaux de Paris, Paris, France; Maria Luisa Azzolini, S Raffaele University Hospital, Milan, Italy; Ronald Bartels, Radboud University Medical Center, Nijmegen, The Netherlands; Pál Barzó, University of Szeged, Szeged, Hungary; Romuald Beauvais, ARTTIC, Munchen, Germany; Ronny Beer, Medical University of Innsbruck, Innsbruck, Austria; Bo-Michael Bellander, Karolinska University Hospital, Stockholm, Sweden; Antonio Belli, NIHR Surgical Reconstruction and Microbiology Research Centre, Birmingham, UK; Habib Benali, Assistance Publique – Hopitaux de Paris, Paris, France; Maurizio Berardino; AOU Città della Salute e della Scienza di Torino - Orthopedic and Trauma Center, Torino, Italy; Luigi Beretta, S Raffaele University Hospital, Milan, Italy; Morten Blaabjerg, Odense University Hospital, Odense, Denmark; Peter Bragge, BehaviourWorks Australia, Monash Sustainability Institute, Monash University, Victoria, Australia; Alexandra Brazinova, Trnava University, Trnava, Slovakia; Vibeke Brinck, Quesgen Systems Inc., Burlingame, CA, USA; Joanne Brooker, Monash University, Melbourne, Australia; Camilla Brorsson, Umeå University, Umeå, Sweden; Andras Buki, University of Pécs, Hungary; Monika Bullinger, Universitätsklinikum Hamburg-Eppendorf, Hamburg, Germany; Manuel Cabeleira, University of Cambridge, Addenbrooke's Hospital, Cambridge, UK; Alessio Caccioppola, Fondazione IRCCS Cà Granda Ospedale Maggiore Policlinico, Milan, Italy; Emiliana Calappi, Fondazione IRCCS Cà Granda Ospedale Maggiore Policlinico, Milan, Italy; Maria Rosa Calvi, S Raffaele University Hospital, Milan, Italy; Peter Cameron, Monash University, Melbourne, Victoria, Australia; Guillermo Carbayo Lozano, Hospital of Cruces, Bilbao, Spain; Marco Carbonara, Fondazione IRCCS Cà Granda Ospedale Maggiore Policlinico, Milan, Italy; Simona Cavallo, AOU Città della Salute e della Scienza di Torino - Orthopedic and Trauma Center, Torino, Italy; Giorgio Chevallard, Niguarda Hospital, Milan, Italy; Arturo Chieregato, Niguarda Hospital, Milan, Italy; Giuseppe Citerio, Università Milano Bicocca, Milano, Italy and ASST di Monza, Monza, Italy; Hans Clusmann, RWTH Aachen University, Aachen, Germany; Mark Coburn, University Hospital Bonn, Bonn, Germany; Jonathan Coles, Cambridge University Hospital NHS Foundation Trust, Cambridge, UK; Jamie D. Cooper, Monash University and The Alfred Hospital, Melbourne, Victoria, Australia; Marta Correia, MRC Cognition and Brain Sciences Unit, Cambridge, UK; Amra Čović, Universitätsmedizin Göttingen, Göttingen, Germany; Nicola Curry, Oxford University Hospitals NHS Trust, Oxford, UK; Endre Czeiter, University of Pécs, Hungary; Marek Czosnyka, University of Cambridge, Addenbrooke's Hospital, Cambridge, UK; Claire Dahyot-Fizelier, CHU Poitiers, Potiers, France; Paul Dark, Salford Royal Hospital NHS Foundation Trust, Salford, UK; Helen Dawes, Oxford Brookes University, Oxford, UK; Véronique De Keyser, Antwerp University Hospital and University of Antwerp, Edegem, Belgium; Vincent Degos, Assistance Publique – Hopitaux de Paris, Paris, France; Francesco Della Corte, Maggiore Della Carità Hospital, Novara, Italy; Hugo den Boogert, Radboud University Medical Center, Nijmegen, The Netherlands; Bart Depreitere, University Hospitals Leuven, Leuven, Belgium; Đula Đilvesi, University of Novi Sad, Novi Sad, Serbia; Abhishek Dixit, University of Cambridge, Addenbrooke's Hospital, Cambridge, UK; Emma Donoghue, Monash University, Melbourne, Australia; Jens Dreier, Charité – Universitätsmedizin Berlin, Germany; Guy-Loup Dulière, CHR Citadelle, Liège, Belgium; Ari Ercole, University of Cambridge, Addenbrooke's Hospital, Cambridge, UK; Patrick Esser, Oxford Brookes University, Oxford, UK; Erzsébet Ezer, University of Pécs, Pécs, Hungary; Martin Fabricius, Region Hovedstaden Rigshospitalet, Copenhagen, Denmark; Valery L. Feigin, National Institute for Stroke and Applied Neurosciences, Auckland University of Technology, Auckland, New Zealand; Kelly Foks, Erasmus MC, Rotterdam, The Netherlands; Shirin Frisvold, University Hospital Northern Norway, Tromso, Norway; Alex Furmanov, Hadassah-Hebrew University Medical Center, Jerusalem, Israel; Pablo Gagliardo, Fundación Instituto Valenciano de Neurorrehabilitación (FIVAN), Valencia, Spain; Damien Galanaud, Assistance Publique – Hopitaux de Paris, Paris, France; Dashiell Gantner, Monash University, Melbourne, Victoria, Australia; Guoyi Gao, Shanghai Renji Hospital, Shanghai Jiaotong University, Shanghai, China; Pradeep George, Karolinska Institutet, Stockholm, Sweden; Alexandre Ghuysen, CHU, Liège, Belgium; Lelde Giga, Pauls Stradins Clinical University Hospital, Riga, Latvia; Ben Glocker, Imperial College London, London, UK; Jagoš Golubovic, University of Novi Sad, Novi Sad, Serbia; Pedro A. Gomez, Hospital Universitario 12 de Octubre, Madrid, Spain; Johannes Gratz, Critical Care and Pain Medicine, Medical University of Vienna, Austria; Benjamin Gravesteijn, Erasmus Medical Center-University Medical Center, Rotterdam, The Netherlands; Francesca Grossi, Maggiore Della Carità Hospital, Novara, Italy; Russell L. Gruen, Australian National University, Canberra, Australia; Deepak Gupta, All India Institute of Medical Sciences, New Delhi, India; Juanita A. Haagsma, Erasmus Medical Center-University Medical Center, Rotterdam, The Netherlands; Iain Haitsma, Erasmus MC, Rotterdam, The Netherlands; Raimund Helbok, Medical University of Innsbruck, Innsbruck, Austria; Eirik Helseth, Oslo University Hospital, Oslo, Norway; Lindsay Horton, University of Stirling, Stirling, UK; Jilske Huijben, Erasmus Medical Center-University Medical Center, Rotterdam, The Netherlands; Peter J. Hutchinson, Addenbrooke's Hospital & University of Cambridge, Cambridge, UK; Bram Jacobs, University of Groningen, University Medical Center Groningen, Groningen, The Netherlands; Stefan Jankowski, Sheffield Teaching Hospitals NHS Foundation Trust, Sheffield, UK; Mike Jarrett, Quesgen Systems Inc., Burlingame, CA, USA; Ji-yao Jiang, Karolinska Institutet, Stockholm, Sweden; Faye Johnson, Salford Royal Hospital NHS Foundation Trust, Salford, UK; Kelly Jones, Auckland University of Technology, Auckland, New Zealand; Mladen Karan, University of Novi Sad, Novi Sad, Serbia; Angelos G. Kolias, Addenbrooke's Hospital & University of Cambridge, Cambridge, UK; Erwin Kompanje, Erasmus Medical Center, Rotterdam, The Netherlands; Daniel Kondziella, Region Hovedstaden Rigshospitalet, Copenhagen, Denmark; Evgenios Kornaropoulos, University of Cambridge, Addenbrooke's Hospital, Cambridge, UK; Lars-Owe Koskinen, Umeå University, Umeå, Sweden; Noémi Kovács, University of Pécs, Pécs, Hungary; Ana Kowark, University Hospital of Aachen, Aachen, Germany; Alfonso Lagares, Hospital Universitario 12 de Octubre, Madrid, Spain; Linda Lanyon, Karolinska Institutet, Stockholm, Sweden; Steven Laureys, University of Liège, Liège, Belgium; Fiona Lecky, University of Sheffield, Sheffield, UK and Salford Royal Hospital, Salford UK; Didier Ledoux, University of Liège, Liège, Belgium; Rolf Lefering, Witten/Herdecke University, Cologne, Germany; Valerie Legrand, ICON, Paris, France; Aurelie Lejeune, Lille University Hospital, Lille, France; Leon Levi, Rambam Medical Center, Haifa, Israel; Roger Lightfoot, University Hospitals Southhampton NHS Trust, Southhampton, UK; Hester Lingsma, Erasmus Medical Center-University Medical Center, Rotterdam, The Netherlands; Andrew I.R. Maas, Antwerp University Hospital and University of Antwerp, Edegem, Belgium; Ana M. Castaño-León, Hospital Universitario 12 de Octubre, Madrid, Spain; Marc Maegele, Cologne-Merheim Medical Center (CMMC), Witten/Herdecke University, Cologne, Germany; Marek Majdan, Trnava University, Trnava, Slovakia; Alex Manara, Southmead Hospital, Bristol, Bristol, UK; Geoffrey Manley, University of California, San Francisco, CA, USA; Costanza Martino, M. Bufalini Hospital, Cesena, Italy; Hugues Maréchal, CHR Citadelle, Liège, Belgium; Julia Mattern, University Hospital Heidelberg, Heidelberg, Germany; Catherine McMahon, The Walton Centre NHS Foundation Trust, Liverpool, UK; Béla Melegh, University of Pécs, Pécs, Hungary; David Menon, University of Cambridge, Addenbrooke's Hospital, Cambridge, UK; Tomas Menovsky, Antwerp University Hospital and University of Antwerp, Edegem, Belgium; Ana Mikolic, Erasmus Medical Center-University Medical Center, Rotterdam, The Netherlands; Benoit Misset, University of Liège, Liège, Belgium; Visakh Muraleedharan, Karolinska Institutet, Stockholm, Sweden; Lynnette Murray, Monash University, Melbourne, Victoria, Australia; Ancuta Negru, Emergency County Hospital Timisoara , Timisoara, Romania; David Nelson, Karolinska Institutet, Stockholm, Sweden; Virginia Newcombe, University of Cambridge, Addenbrooke's Hospital, Cambridge, UK; Daan Nieboer, Erasmus Medical Center-University Medical Center, Rotterdam, The Netherlands; József Nyirádi, University of Pécs, Pécs, Hungary; Otesile Olubukola, University of Sheffield, Sheffield, UK; Matej Oresic, Örebro University, Örebro, Sweden; Fabrizio Ortolano, Fondazione IRCCS Cà Granda Ospedale Maggiore Policlinico, Milan, Italy; Aarno Palotie, University of Helsinki, Helsinki, Finland, Massachusetts General Hospital, Boston, MA, USA, and The Broad Institute of MIT and Harvard, Cambridge, MA, USA; Paul M. Parizel, University of Antwerp, Edegem, Belgium; Jean-François Payen, University Hospital of Grenoble, Grenoble, France; Natascha Perera, ARTTIC, Munchen, Germany; Vincent Perlbarg, Assistance Publique – Hopitaux de Paris, Paris, France; Paolo Persona, Azienda Ospedaliera Università di Padova, Padova, Italy; Wilco Peul, Leiden University Medical Center, Leiden, The Netherlands and Medical Center Haaglanden, The Hague, The Netherlands; Anna Piippo-Karjalainen, Helsinki University Central Hospital; Matti Pirinen, University of Helsinki, Helsinki, Finland; Dana Pisica, Erasmus Medical Center-University Medical Center, Rotterdam, The Netherlands; Horia Ples, Emergency County Hospital Timisoara, Timisoara, Romania; Suzanne Polinder, Erasmus Medical Center-University Medical Center, Rotterdam, The Netherlands; Inigo Pomposo, Hospital of Cruces, Bilbao, Spain; Jussi P. Posti, Turku University Hospital and University of Turku, Turku, Finland; Louis Puybasset, Pitié -Salpêtrière Teaching Hospital, Assistance Publique, Hôpitaux de Paris and University Pierre et Marie Curie, Paris, France; Andreea Radoi, Vall d'Hebron Research Institute, Barcelona, Spain; Arminas Ragauskas, Kaunas University of Technology and Vilnius University, Vilnius, Lithuania; Rahul Raj, Helsinki University Central Hospital; Malinka Rambadagalla, Rezekne Hospital, Latvia; Isabel Retel Helmrich, Erasmus Medical Center-University Medical Center, Rotterdam, The Netherlands; Jonathan Rhodes, NHS Lothian & University of Edinburg, Edinburgh, UK; Sylvia Richardson, Cambridge Institute of Public Health, Cambridge, UK; Sophie Richter, University of Cambridge, Addenbrooke's Hospital, Cambridge, UK; Samuli Ripatti, University of Helsinki, Helsinki, Finland; Saulius Rocka, Kaunas University of Technology and Vilnius University, Vilnius, Lithuania; Cecilie Roe, Oslo University Hospital/University of Oslo, Oslo, Norway; Olav Roise, Oslo University Hospital/University of Oslo, Oslo, Norway; Jonathan Rosand, Broad Institute, Cambridge MA, USA, Harvard Medical School, Boston MA, USA, and Massachusetts General Hospital, Boston MA, USA; Jeffrey V. Rosenfeld, The Alfred Hospital, Monash University, Melbourne, Victoria, Australia; Christina Rosenlund, Odense University Hospital, Odense, Denmark; Guy Rosenthal, Hadassah-Hebrew University Medical Center, Jerusalem, Israel; Rolf Rossaint, University Hospital of Aachen, Aachen, Germany; Sandra Rossi, Azienda Ospedaliera Università di Padova, Padova, Italy; Daniel Rueckert, Imperial College London, London, UK; Martin Rusnák, International Neurotrauma Research Organisation, Vienna, Austria; Juan Sahuquillo, Vall d'Hebron Research Institute, Barcelona, Spain; Oliver Sakowitz, University Hospital Heidelberg, Heidelberg, Germany and Klinik für Neurochirurgie, Klinikum Ludwigsburg, Ludwigsburg, Germany; Renan Sanchez-Porras, Klinik für Neurochirurgie, Klinikum Ludwigsburg, Ludwigsburg, Germany; Janos Sandor, University of Debrecen, Debrecen, Hungary; Nadine Schäfer, Witten/Herdecke University, Cologne, Germany; Silke Schmidt, University Greifswald, Greifswald, Germany; Herbert Schoechl, UVA Trauma Hospital, Salzburg, Austria; Guus Schoonman, Elisabeth-TweeSteden Ziekenhuis, Tilburg, The Netherlands; Rico Frederik Schou, Odense University Hospital, Odense, Denmark; Elisabeth Schwendenwein, Medical University Vienna, Vienna, Austria; Charlie Sewalt, Erasmus Medical Center-University Medical Center, Rotterdam, The Netherlands; Ranjit D. Singh, Leiden University Medical Center, Leiden, The Netherlands and Medical Center Haaglanden, The Hague, The Netherlands; Toril Skandsen, Norwegian University of Science and Technology, NTNU, Trondheim, Norway and St. Olavs Hospital, Trondheim University Hospital, Trondheim, Norway; Peter Smielewski, University of Cambridge, Addenbrooke's Hospital, Cambridge, UK; Abayomi Sorinola, University of Pécs, Pécs, Hungary; Emmanuel Stamatakis, University of Cambridge, Addenbrooke's Hospital, Cambridge, UK; Simon Stanworth, Oxford University Hospitals NHS Trust, Oxford, UK; Robert Stevens, John Hopkins University School of Medicine, Baltimore, USA; William Stewart, Queen Elizabeth University Hospital and University of Glasgow, Glasgow, UK; Ewout W. Steyerberg, Erasmus Medical Center-University Medical Center, Rotterdam, The Netherlands and Leiden University Medical Center, Leiden, The Netherlands; Nino Stocchetti, Milan University and Fondazione IRCCS Cà Granda Ospedale Maggiore Policlinico, Milano, Nina Sundström, Umeå University, Umeå, Sweden; Riikka Takala, Turku University Hospital and University of Turku, Turku, Finland; Viktória Tamás, University of Pécs, Pécs, Hungary; Tomas Tamosuitis, Kaunas University of Health Sciences, Kaunas, Lithuania; Mark Steven Taylor, Trnava University, Trnava, Slovakia; Braden Te Ao, Auckland University of Technology, Auckland, New Zealand; Olli Tenovuo, Turku University Hospital and University of Turku, Turku, Finland; Alice Theadom, Auckland University of Technology, Auckland, New Zealand; Matt Thomas, Southmead Hospital, Bristol, Bristol, UK; Dick Tibboel, Erasmus Medical Center, Sophia Children's Hospital, Rotterdam, The Netherlands; Marjolein Timmers, Erasmus Medical Center, Rotterdam, The Netherlands; Christos Tolias, Kings College London, London, UK; Tony Trapani, Monash University, Melbourne, Victoria, Australia; Cristina Maria Tudora, Emergency County Hospital Timisoara, Timisoara, Romania; Andreas Unterberg, University Hospital Heidelberg, Heidelberg, Germany; Peter Vajkoczy, Charité – Universitätsmedizin Berlin, Berlin, Germany; Shirley Vallance, Monash University, Melbourne, Victoria, Australia; Egils Valeinis, Pauls Stradins Clinical University Hospital, Riga, Latvia; Zoltán Vámos, University of Pécs, Pécs, Hungary; Mathieu van der Jagt, Erasmus MC– University Medical Center Rotterdam, Rotterdam, The Netherlands; Gregory Van der Steen, Antwerp University Hospital and University of Antwerp, Edegem, Belgium; Joukje van der Naalt, University of Groningen, University Medical Center Groningen, Groningen, The Netherlands; Jeroen T.J.M. van Dijck, Leiden University Medical Center, Leiden, The Netherlands and Medical Center Haaglanden, The Hague, The Netherlands; Inge A. van Erp, Leiden University Medical Center, Leiden, The Netherlands and Medical Center Haaglanden, The Hague, The Netherlands; Thomas A. van Essen, Leiden University Medical Center, Leiden, The Netherlands and Medical Center Haaglanden, The Hague, The Netherlands; Wim Van Hecke, icoMetrix NV, Leuven, Belgium; Caroline van Heugten, Oxford Brookes University, Oxford, UK; Dominique Van Praag, Antwerp University Hospital, Edegem, Belgium; Ernest van Veen, Erasmus Medical Center-University Medical Center, Rotterdam, The Netherlands; Thijs Vande Vyvere, icoMetrix NV, Leuven, Belgium; Roel P. J. van Wijk, Leiden University Medical Center, Leiden, The Netherlands and Medical Center Haaglanden, The Hague, The Netherlands; Alessia Vargiolu, ASST di Monza, Monza, Italy; Emmanuel Vega, Lille University Hospital, Lille, France; Kimberley Velt, Erasmus Medical Center-University Medical Center, Rotterdam, The Netherlands; Jan Verheyden, icoMetrix NV, Leuven, Belgium; Paul M. Vespa, University of California, Los Angeles, CA, USA; Anne Vik, Norwegian University of Science and Technology, NTNU, Trondheim, Norway; Rimantas Vilcinis, Kaunas University of Health Sciences, Kaunas, Lithuania; Victor Volovici, Erasmus MC, Rotterdam, The Netherlands; Nicole von Steinbüchel, Universitätsmedizin Göttingen, Göttingen, Germany; Daphne Voormolen, Erasmus Medical Center-University Medical Center, Rotterdam, The Netherlands; Petar Vulekovic, University of Novi Sad, Novi Sad, Serbia; Kevin K.W. Wang, University of Florida, Gainesville, FL, USA; Daniel Whitehouse, University of Cambridge, Addenbrooke's Hospital, Cambridge, UK; Eveline Wiegers, Erasmus Medical Center-University Medical Center, Rotterdam, The Netherlands; Guy Williams, University of Cambridge, Addenbrooke's Hospital, Cambridge, UK; Lindsay Wilson, University of Stirling, Stirling, UK; Stefan Winzeck, University of Cambridge, Addenbrooke's Hospital, Cambridge, UK; Stefan Wolf, Charité – Universitätsmedizin Berlin, Germany; Zhihui Yang, Broad Institute, Cambridge MA, USA, Harvard Medical School, Boston MA, USA, and Massachusetts General Hospital, Boston MA, USA; Peter Ylén, VTT Technical Research Centre, Tampere, Finland; Alexander Younsi, University Hospital Heidelberg, Heidelberg, Germany; Frederick A. Zeiler, University of Cambridge, Addenbrooke's Hospital, Cambridge, UK and University of Manitoba, Winnipeg, MB, Canada; Veronika Zelinkova, Trnava University, Trnava, Slovakia; Agate Ziverte, Pauls Stradins Clinical University Hospital, Riga, Latvia; Tommaso Zoerle, Fondazione IRCCS Cà Granda Ospedale Maggiore Policlinico, Milan, Italy.
