# Supplementary material for: Rehabilitation and outcomes after complicated vs uncomplicated mild TBI: results from the CENTER-TBI study
Source: BMC Health Serv Res. 2022 Dec 16;22:1536. doi: 10.1186/s12913-022-08908-0 (PMC9758851; doi:10.1186/s12913-022-08908-0)
Supplement: Supplementary file 4 — Additional file 4. Sites, ethical committees, and ethical approval. [file 12913_2022_8908_MOESM4_ESM.pdf]

Additional file 4 - Sites, ethical committees, and ethical approval

Country	City	Insitute	Ethical Commitee	Number Approval	Date Approval
**Austria**	Vienna	Medizinische Universität Wien<br>Universitätsklinik für Unfallchirurgie	Ethikkommission der Medizinischen Universität Wien	1646/2014	3/05/2016
**Austria**	Innsbruck	Medizinische Universität Innsbruck<br>Universitätsklinik für Neurologie	Ethikkommission der Medizinischen Universität Innsbruck	AN2014-0336 343/4.22	1/12/2014
**Belgium**	Antwerp	Antwerp University Hospital	Centraal Ethisch Comité - Ethisch Comité Universitair Ziekenhuis Antwerpen en de Universiteit Antwerpen	B300201422714	17/11/2014
**Belgium**	Liège	CHR Citadelle	- Centraal Ethisch Comité - Ethisch Comité Universitair Ziekenhuis Antwerpen en de Universiteit Antwerpen<br>- Comité d'Ethique 412	- B300201422714<br>- 1427	-17/11/2014<br>- 25/11/2014
**Belgium**	Liège	CHU	- Centraal Ethisch Comité - Ethisch Comité Universitair Ziekenhuis Antwerpen en de Universiteit Antwerpen<br>- Comité d'Ethique hospitalo-facultaire niversitaire de Liège (707)	- B300201422714<br>- B707201422102 / 2014-244	- 17/11/2014<br>- 07/10/2014
**Belgium**	Leuven	UZ Leuven	- Centraal Ethisch Comité - Ethisch Comité Universitair Ziekenhuis Antwerpen en de Universiteit Antwerpen<br>- Comissie Medische Ethiek UZ KU Leuven / Onderzoek	- B300201422714<br>- B322201523981 / S57019 (ML11365)	- 06/13/2015<br>- 07/10/2014
**Denmark**	Odense	Odense Universiteitshospital - Neurokirurgisk afdeling	De Videnskabsetiske Komitéer for Region Syddanmark	S-20140215	16/03/2015
**Denmark**	Copenhagen	Region Hovedstaden Rigshopitalet	De Videnskabsetiske Komitéer for Region Syddanmark	S-20140215	16/03/2015
**Finland**	Turku	Turku University Hospital	Varsinais suomen sairaanhoitopiirin kuntayhtyma - Eettinen Toimikunta	95/1801/2014	24/10/2014
**Finland**	Helsinki	Helsinki University Central Hospital	Varsinais suomen sairaanhoitopiirin kuntayhtyma - Eettinen Toimikunta	95/1801/2014	24/10/2014
**France**	Paris	APHP	Agence Nationale de Sécurité du Médicament et des Produits de Santé ANSM	141421B-31	19/12/2014
**France**	Besançon	CHRU de Besançon	Agence Nationale de Sécurité du Médicament et des Produits de Santé ANSM	141421B-31	19/12/2014
**France**	Lille	Lille University Hospital	Agence Nationale de Sécurité du Médicament et des Produits de Santé ANSM	141421B-31	19/12/2014
**France**	Grenoble	University Hospital of Grenoble	Agence Nationale de Sécurité du Médicament et des Produits de Santé ANSM	141421B-31	19/12/2014
**France**	Nancy	University Hospital Nancy	Agence Nationale de Sécurité du Médicament et des Produits de Santé ANSM	141421B-31	19/12/2014
**France**	Poitiers	CHU Poitiers	Agence Nationale de Sécurité du Médicament et des Produits de Santé ANSM	141421B-31	19/12/2014
**Germany**	Heidelberg	Universitätsklinikum Heidelberg<br>Neurochirurgische Kliniki	Ethikkommission Medizinsche Fakultät Heidelberg	S-435/2014	27/05/2015
**Germany**	Berlin	Charité | Campus Virchow Klinikum	Ethikkommission an der Medizinsche Fakultät<br>Der rheinsch-Westfälischen Technischen Hocgschule Aachen	1098/15	26/02/2016
**Germany**	Aachen	Uniklinik RWTH Aachen	Ethikkommission an der Medizinsche Fakultät<br>Der rheinsch-Westfälischen Technischen Hocgschule Aachen	EK 174/15	2/07/2015
**Germany**	Ludwigsburg	Klinikum Ludwigsburg	Ethikkommission Medizinsche Fakultät Heidelberg	S-435/2014	29/01/2016
**Hungary**	Pecs	Pecsi Tudomanyegyetem Klinikai Kozpont	- ETT TUKEB Egészségügyi Tudományos Tanács<br>- Pécsi Tudományegyetem	- 42558-3/2014/EKU<br>- 5421	- 2/10/2014<br>- 28/03/2015
**Hungary**	Szeged	zegedi tudományegyetem orvostudományi kar<br>Szent Györgyi Albert Klinikai Központ	-ETT TUKEB Egészségügyi Tudományos Tanács<br>- Szegedi Tudományegyetem	- 42558-3/2014/EKU<br>- 3803	- 2/10/2014<br>- 23/05/2016
**Israel**	Haifa	Rambam Medical Center	Helsinki Committee, Rambam Health Care Campus	RMB 373-14	22/07/2015
**Israel**	Jeruzalem	Hadassah-hebrew University Medical Center	Hadassah Medical Organization IRB	0590-16 HMO	6/12/2016
**Italy**	Milan	Fondazione IRCCS Ca' Granda Ospedale Maggiore Policlinico	Fondazione IRCCS Ca' Granda Ospedale Maggiore Policlinico - Direzione Scientifica Comitato Etico	542/2014	20/10/2014
**Italy**	Milan	Ospedale San Raffaele	Comitato Etico - Ospedale San Raffaele	217/2014	10/04/2015
**Italy**	Torino	AOU Città della Salute e della Scienza di Torino	Comitato Etico Interaziendale A.O.U. Città della Salute e della Scienza di Torino - A.O. Ordine Mauriziano - A.S.L.	0015269	15/02/2016
**Italy**	Cesena	Bufalini Hospital	Comitato Etico IRST IRCCS AVR	1675/2015 I.5/207	18/03/2015
**Italy**	Padova	Azienda Ospedaliera Università di Padova	Comitato Etico - Ospedale San Raffaele	217/2014	10/04/2015
**Italy**	Monza	San Gerardo Hospital/ASST	Comitato Etico Della Provincia Monza Brianza	1978/2014	22/12/2014
**Italy**	Novara	Maggiore Della Carità Hospital	Comitato Etico Interaziendale A.O.U. 'Maggiore della Carità'	CE 46/15	3/07/2015
**Italy**	Milan	Niguarda Hospital	Comitato Etico - Ospedale Niguarda Ca' Granda	636-122015	23/12/2015
**Latvia**	Riga	Pauls Stradins Clinical University Hospital	Ethics Commiitee for Clinical Research at Pauls Stradins Clinical University Hospital Development Society	171215-1E	17/12/2015
**Latvia**	Riga	Riga Eastern Clinical University Hospital	Ethics Commiitee for Clinical Research at Pauls Stradins Clinical University Hospital Development Society	171215-1E	17/12/2015
**Latvia**	Rezekne	Rezekne Hospital	Ethics Commiitee for Clinical Research at Pauls Stradins Clinical University Hospital Development Society	171215-1E	17/12/2015
**Lithuania**	Vilnius	Vilniaus Universiteto Ligonine	VILNIAUS REGIONINIS BIOMEDICININIŲ TYRIMŲ ETIKOS KOMITETAS	158200-15-801-323	6/10/2015
**Lithuania**	Kaunas	LSMUL Kauno klinikos Skubio pagalbos skyrius	KAUNO REGIONINIS BIOMEDICININIŲ TYRIMŲ ETIKOS KOMITETAS	BE-2-6	6/01/2015
**Netherlands**	Leiden	Het Leids Universitair Medisch Centrum te Leiden	Leids Universitair Centrum - Commissie Medische Ethiek	P14.222/NV/nv	3/12/2014
**Netherlands**	Rotterdam	Erasmus MC	Leids Universitair Centrum - Commissie Medische Ethiek	P14.222/NV/nv	3/12/2014
**Netherlands**	The Hague	Medisch Centrum Haaglanden	Leids Universitair Centrum - Commissie Medische Ethiek	P14.222/NV/nv	3/12/2014
**Netherlands**	The Hague	Het Haga Hospital	Leids Universitair Centrum - Commissie Medische Ethiek	P14.222/NV/nv	25/11/2015
**Netherlands**	Nijmegen	Radboud UMC	Leids Universitair Centrum - Commissie Medische Ethiek	P14.222/NV/nv	2/07/2015
**Netherlands**	Tilburg	St. Elisabeth Ziekenhuis	Leids Universitair Centrum - Commissie Medische Ethiek	P14.222/NV/nv	2/07/2015
**Netherlands**	Groningen	UMC Groningen	Leids Universitair Centrum - Commissie Medische Ethiek	P14.222/NV/nv	3/12/2014
**Norway**	Tromso	Universitetssykehuset Nord-Norge	Regional komité for medisinsk og helsefaglig<br>forskningsetikk REK midt-Norge (REK midt)	2014/1454	23/03/2015
**Norway**	Trondheim	St.Olavs Hospital	Regional komité for medisinsk og helsefaglig<br>forskningsetikk REK midt-Norge (REK midt)	2014/1454	23/03/2015
**Norway**	Oslo	Oslo Universitetssykehus	Regional komité for medisinsk og helsefaglig<br>forskningsetikk REK midt-Norge (REK midt)	2014/1454	23/03/2015
**Romania**	Timisoara	Clinica de Neurochirurgie<br>Universitatea de Medicina si Farmacie "Victor Babes" Timisoara	Comitetului de Etica a Spitalului Clinic Judeteam de Urgenta Timisoara	73	16/10/2014
**Serbia**	Novi Sad	Klinidkog centra Vojvodine	Etidkog odbora Klinidkog centra Vojvodine	00-08/332	11/07/2014
**Spain**	Madrid	Hospital Universitario 12 de Octubre	Comité Etico de Investigacion Clinica del Hospital Universitario 12 de Octubre	14/262	3/11/2014
**Spain**	Barcelona	Vall d'Hebron University Hospital	Comité ético de investigación clínica y comisión de proyectos de investigación del hospital universitari Vall d'Hebron	ID-RTF080	13/11/2014

**Spain**	Bilbao	Clínico Universitario de Cruces	Comité Etico de Investigacion Clinica de Euskadi	PI2014158	24/02/2015
**Spain**	Valencia	Clínico Universitario de Valencia	Comité Etico de Investigacion Clinica del Clínico Universitario de Valencia	F-CE-GEva-15	23/04/2015
**Sweden**	Stockholm	Karolinksa University Hospital	EPN (Regionala Etikprövningsnämnden i Stockholm)	2014/1473-31/4	24/09/2014
**Sweden**	Umea	Umea University Hosptital	EPN (Regionala Etikprövningsnämnden i Stockholm)	2014/1473-31/4	24/09/2014
**Switzerland**	Lausanne	Centre hospitalier universitaire Vaudois	La Commission cantonale (VD) d'éthique de la recherche sur l'être humain (CER-VD)	473/11	9/12/2014
**UK**	Birmingham	Queen Elizabeth Hospital	- NHS HRA<br>- UHB Research Governance Office - Queen Elizabeth Hospital	- 14/SC/1370<br>- RRK5224	- 22/12/2014<br>- 24/02/2016
**UK**	Cambridge	Cambridge University Hospital NHS Foundation Trust	- NHS HRA<br>- Research and Development Department - Cambridge University Hospital NHS Foundation Trust	- 14/SC/1370<br>- AO93184	- 22/12/2014<br>- 30/01/2015
**UK**	Southampton	University Hospitals Southhampton NHS Trust	- NHS HRA<br>- Research Governance Office - University Hospitals Southhampton NHS Trust	- 14/SC/1370<br>- RHM CRI0294	- 22/12/2014<br>- 13/03/2015
**UK**	Sheffield	Sheffield Teaching Hospitals NHS Foundation Trust	- NHS HRA<br>- Research and Development Department -Sheffield Teaching Hospitals NHS Foundation Trust	- 14/SC/1370<br>- STH18187	- 22/12/2014<br>- 25/03/2015
**UK**	London	Kings college London	- NHS HRA<br>- Research & Innovation Office - Kings college London NHS Foundation Trust	- 14/SC/1370<br>- KCH15-204	- 22/12/2014<br>- 30/12/2015
**UK**	Salford	Salford Royal Hospital	- NHS HRA<br>- Research and Development Department -Salford Royal Hospital NHS Foundation Trust	- 14/SC/1370<br>- 2015/025ET	- 22/12/2014<br>- 27/07/2015
**UK**	Liverpool	The Walton centre NHS Foundation Trust	- NHS HRA<br>- Research & Innovation Office - The Walton centre NHS Foundation Trust	- 14/SC/1370<br>- RG154-15	- 22/12/2014<br>- 11/05/2015
**UK**	Bristol	Southmead Hospital Bristol	- NHS HRA<br>- Research & Innovation - North Bristol NHS Trust	- 14/SC/1370<br>- 3427	- 22/12/2014<br>- 23/12/2014
**UK/Scotland**	Edinburgh	Lothian Health Board	- NHS Scotland<br>- Research and Development Department - University Hospitals Division NHS Lothian	- 14/SS/1086<br>- 2015/0171	- 23/12/2014<br>- 19/06/2015
